# A neural network to create super‐resolution MR from multiple 2D brain scans of pediatric patients

**DOI:** 10.1002/mp.17563

**Published:** 2024-12-10

**Authors:** Jose Benitez‐Aurioles, Eliana M. Vásquez Osorio, Marianne C. Aznar, Marcel Van Herk, Shermaine Pan, Peter Sitch, Anna France, Ed Smith, Angela Davey

**Affiliations:** ^1^ Division of Informatics, Imaging and Data Sciences University of Manchester Manchester UK; ^2^ Radiotherapy‐Related Research Group, Division of Cancer Sciences, School of Medical Sciences, Faculty of Biology, Medicine and Health University of Manchester Manchester UK; ^3^ The Christie NHS Foundation Trust Manchester UK

**Keywords:** machine learning, pediatric oncology, super‐resolution

## Abstract

**Background:**

High‐resolution (HR) 3D MR images provide detailed soft‐tissue information that is useful in assessing long‐term side‐effects after treatment in childhood cancer survivors, such as morphological changes in brain structures. However, these images require long acquisition times, so routinely acquired follow‐up images after treatment often consist of 2D low‐resolution (LR) images (with thick slices in multiple planes).

**Purpose:**

In this work, we present a super‐resolution convolutional neural network, based on previous single‐image MRI super‐resolution work, that can reconstruct a HR image from 2D LR slices in multiple planes in order to facilitate the extraction of structural biomarkers from routine scans.

**Methods:**

A multilevel densely connected super‐resolution convolutional neural network (mDCSRN) was adapted to take two perpendicular LR scans (e.g., coronal and axial) as tensors and reconstruct a 3D HR image. A training set of 90 HR T1 pediatric head scans from the Adolescent Brain Cognitive Development (ABCD) study was used, with 2D LR images simulated through a downsampling pipeline that introduces motion artifacts, blurring, and registration errors to make the LR scans more realistic to routinely acquired ones.

The outputs of the model were compared against simple interpolation in two steps. First, the quality of the reconstructed HR images was assessed using the peak signal‐to‐noise ratio and structural similarity index compared to baseline. Second, the precision of structure segmentation (using the autocontouring software Limbus AI) in the reconstructed versus the baseline HR images was assessed using mean distance‐to‐agreement (mDTA) and 95% Hausdorff distance. Three datasets were used: 10 new ABCD images (dataset 1), 18 images from the Children's Brain Tumor Network (CBTN) study (dataset 2) and 6 “real‐world” follow‐up images of a pediatric head and neck cancer patient (dataset 3).

**Results:**

The proposed mDCSRN outperformed simple interpolation in terms of visual quality. Similarly, structure segmentations were closer to baseline images after 3D reconstruction. The mDTA improved to, on average (95% confidence interval), 0.7 (0.4–1.0) and 0.8 (0.7–0.9) mm for datasets 1 and 3 respectively, from the interpolation performance of 6.5 (3.6–9.5) and 1.2 (1.0–1.3) mm.

**Conclusions:**

We demonstrate that deep learning methods can successfully reconstruct 3D HR images from 2D LR ones, potentially unlocking datasets for retrospective study and advancing research in the long‐term effects of pediatric cancer. Our model outperforms standard interpolation, both in perceptual quality and for autocontouring. Further work is needed to validate it for additional structural analysis tasks.

## 1 INTRODUCTION

Tumors in the brain, central nervous system, or head‐and‐neck account for over a third of all cancer diagnoses each year in adults,^1^ and over a quarter in children.^2^, ^3^ For many pediatric patients, radiotherapy will form an important part of treatment. Due to their long lifespan after treatment, childhood cancer survivors are at high risk of experiencing long‐term side‐effects. For example, irradiation of the brain may lead to cognitive and endocrine dysfunction.^4^, ^5^, ^6^, ^7^ Many side effects are associated with structural changes in anatomy over time.^8^, ^9^ The segmentation or longitudinal registration of brain structures would allow for quantitative tracking of these changes, providing insight in the relationship between side‐effects, multi‐modality treatment, radiotherapy dose and patient characteristics.^10^, ^11^

To properly segment brain structures, high‐quality 3D imaging is required. Magnetic resonance imaging (MRI) is particularly useful for this task, due to its good soft tissue contrast.^12^ MRI is also frequently used in the routine follow‐up of cancer survivors, to assess potential relapse.^13^ However, the acquisition of high‐resolution (HR) scans can be time consuming, thereby increasing its cost and the risk of patient motion artifacts in the final image,^13^, ^14^, ^15^ especially for children. As a result, practitioners often choose to perform faster imaging sequences, that is, 2D slices in different intervals and directions. Slice distance and thickness are typically much larger than their in‐plane resolution. This type of low‐resolution (LR) is especially common in pediatric patients and fetal imaging, where voluntary motion can be difficult or impossible to control.^16^, ^17^, ^18^ Although these images are appropriate for routine follow‐up of the patient after treatment, their anisotropic resolution is an obstacle for retrospective studies that investigate the evolution of structural biomarkers over time. For example, it has been suggested that hippocampal volume is affected by radiation dose, and volume changes could be used as a marker of cognitive decline.^19^

For this reason, researchers have attempted to increase the resolution of 2D MRI scans post‐hoc. Single‐image super‐resolution methods have been developed and adapted to combine multiple views of a patient for the reconstruction of HR isotropic images. To do this, these models make use of either interpolation or regularized reconstruction.^20^, ^21^, ^22^ While the latter are more accurate, they are, as most model‐based optimization problems, slow and computationally expensive,^23^ making their implementation difficult for large datasets. In addition, reconstruction methods requiring the “raw” magnetic resonance (MR) data, which are generally deleted soon after acquisition in clinics, are in general not available in retrospective studies of routine data. Interpolation is considered a practical approach, but it can be difficult to recover detailed morphological information of brain and facial structures, especially for large sampling distances and slice thicknesses.

To increase efficiency and accuracy, deep learning is being explored for multiple view reconstruction in 3D MRI. This is partly due to the success of convolutional neural networks in isotropic single‐image MRI super‐resolution tasks. Convolutional architectures like U‐Net,^24^, ^25^ Residual Net,^26^ or Dense Net^27^ have been successful in improving the resolution of scans, in both two and three dimensions, as well as dynamic scans. For multi‐image reconstruction, Ebner et al.^28^ developed a slice registration method informed by segmentation based on a neural network. Methods using deep learning as the main component of the reconstruction pipeline improved the visual and structural quality when compared to interpolation methods.^29^, ^30^ However, there is not validation of this approach on non‐simulated images of patients, and the potential of deep learning to improve image resolution of pediatric head scans for structural analysis has not been explored.

In this paper, we aim to build a reconstruction model that combines LR slices in different orientations into a HR 3D image. We assessed the performance of our super‐resolution method by comparing it to an interpolation fusion pipeline, measuring its ability to recover both visual and structural quality using perceptual metrics and a contouring task. The main contributions of this paper are:

1. We adapted a single input super‐resolution dense neural network to multiple inputs from pediatric MRI head scans, training it through a self‐supervised downsampling pipeline with extensive data augmentation;
2. We assessed the ability of our model icn improving structural analysis and biomarker extraction in the brain on routinely acquired MRI data, comparing our method's performance to linear interpolation.

## 2 METHODS

### 2.1 Datasets

The images used to train the network were 80 T1‐weighted MRI scans from the ABCD study,^31^ selected randomly from the 11 000 scans available from healthy children aged 9–10 years old. The images were acquired on multiple scanners from different manufacturers, and the files were raw instead of normalized.^32^ These scans had an isotropic resolution of 1 mm x 1 mm x 1 mm, and dimensions of 256 × 256 × 256 voxels. Ten additional images from the ABCD study were used for hyperparameter optimization.

To assess the performance of the model, we used three datasets:

**Dataset 1**: Ten additional scans from the ABCD study (with the same conditions as the training images) that were not part of the training dataset.

**Dataset 2**: 18 HR T1‐weighted scans acquired in routine practice. Seven of these images were of children with brain tumors (a combination of pre and post‐surgery) obtained from the Children's Brain Tumor Network (CBTN).^33^ The remaining 11 images were of three children treated with proton or photon radiotherapy for head‐and‐neck and brain tumors at the Christie National Health Service (NHS) Foundation Trust. The images consisted of both pre and post‐radiotherapy scans at different timepoints. Institutional approval was granted to access data collected in the UK Computed Aided Theragnostics system^34^ (Application Number: 2022‐010) and data curated by the Proton Clinical Outcome Unit^35^ (Application Number: 2021‐002).

**Dataset 3**: This contained a subset of images from Dataset 2 which represented six approximately yearly follow‐up images of a single patient treated with proton radiotherapy for a head‐and‐neck tumor across multiple timepoints with both acquired HR and acquired LR scans. This subset was chosen as a single example of performance on follow‐up, with these images being the only ones for which there was: (1) a combination of HR images that corresponded to two or more LR ones taken on the same day and; (2) a field of view in these images that would allow for the segmentation of the eyes, the hippocampi, the brainstem, and the optic nerves.

More detail on the dataset's characteristics, including resolution, age, and number of images is available in Table 1.

**TABLE 1:** Summary of the training and three validation datasets.

					High‐resolution scans	Low‐resolution scans (2D axial and coronal)	
Dataset label (*N*)	Data source	Description	Age range (Years)	Sequence	Isotropic resolution (mm)	In‐plane resolution (mm)	Through‐plane resolution (mm)
Training dataset (*N* = 90)	ABCD	Healthy children	9–10	T1w	1.00 (0.00)	1.00 (0.00)	Simulated 3–12 mm
Validation dataset 1 (*N* = 10)	ABCD	Healthy children	9–10	T1w	1.00 (0.00)	1.00 (0.00)	
Validation dataset 2 (*N* = 18)	CBTN	Patients with brain tumors, before and after operation	2–20	T1w	0.72 (0.25)	0.72 (0.25)	
	PCOU	Patients with head and neck tumors, before and after proton therapy.					
	ukCAT	Patients with head and neck tumors, before and after radiotherapy.					
Validation Dataset 3 (*N* = 6)	PCOU	Follow up of one head and neck cancer pediatric patient, after proton therapy.	14–19	T1w	0.70 (0.31)	0.69 (0.04)	8.30 (0.41)

*Note*: Standard deviations are shown in brackets.

Abbreviations: ABCD, adolescent brain cognitive development; CBTN, children's brain tumor network.

### 2.2 Simulating LR from HR images

Paired HR and LR scans of a patient's head are difficult to obtain in large numbers because the circumstances that encourage the acquisition of LR images discourage the acquisition of HR ones. Additionally, in order for a trained super‐resolution model to be agnostic to through‐plane sampling distance and slice thickness, a much larger range of images would be needed to explore the full data distribution. Because of this, we simulated our training set using only HR images. This approach is common to tackle super‐resolution as an unsupervised learning problem.^25^, ^26^, ^36^

To generate anisotropic LR scans from an isotropic HR image, HR images were downsampled in two perpendicular directions, with randomly chosen slice thicknesses of $t_{1,2}∼\mathrm{Uniform}\left(3\;\mathrm{and}\;8\;\mathrm{mm}\right)$ and sampling distances of $d_{1,2}∼\mathrm{Uniform}\left(t_{1,2},t_{1,2}+4\;\mathrm{mm}\right)$. Downsampling was done by first applying unidirectional Gaussian blur with standard deviation $t_{1,2}$. Second, slices were sampled from the resulting blurred scan with separation distances of $d_{1,2}$. This generates two perpendicular LR views of the HR scan, $X_{1}$ and $X_{2}$.

LR scans obtained in real scenarios are not perfectly downsampled representations of the HR images. To make the simulated images more realistic, multiple artifacts, and inconsistencies were introduced. This includes differences in the acquisition sequences,^37^, ^38^ blurring, misregistration artifacts due to the patient's motion,^14^ misregistration between the pairs of LR scans, noise, and random masking of one of the views. In addition, to improve the generalizability of the model across different scanners and acquisition sequences,^37^, ^38^ the brightness and contrast of the images was changed randomly.

Due to the misregistration between the two views, and the misregistration between slices within each view due to motion artifacts, four distinct HR views of the same image, $y_{i1}$, $y_{i2}$, $y_{i3,}$ and $y_{i4}$, were used to create the simulated LR images $X_{1}$ and $X_{2}$. The data augmentation steps were done using the Python package Torchio.^39^

### 2.3 Generating super‐resolution images

We implement a mDCSRN.^27^ This is a 3D convolutional neural network, previously used for isotropic MRI super‐resolution tasks, for which each layer has access to any feature outputted by previous layers. With this architecture, the model has simultaneous access to its low‐level and high‐level features when reconstructing the HR image.^40^ This design is particularly suitable for super‐resolution tasks, as its aim is to reconstruct low‐level features with additional context given by high‐level ones.

We have adapted the mDCSRN model to take as input two perpendicular image stacks of the same image region, $X_{1}$ and $X_{2}$. The two stacks, represented by 3D tensors, are concatenated into a 4D tensor after being upsampled through linear interpolation to the target resolution of 1mm×1mm×1mm. This choice allows the network to be agnostic to original sampling distance and slice thickness. Due to the computational requirements of training 3D convolutional neural networks, the input, and output are set to a fixed size of 40×40×40mm images. Scans beyond this size were decomposed into smaller patches, and their outputs were then aggregated back to its original size.

**FIGURE 1:**
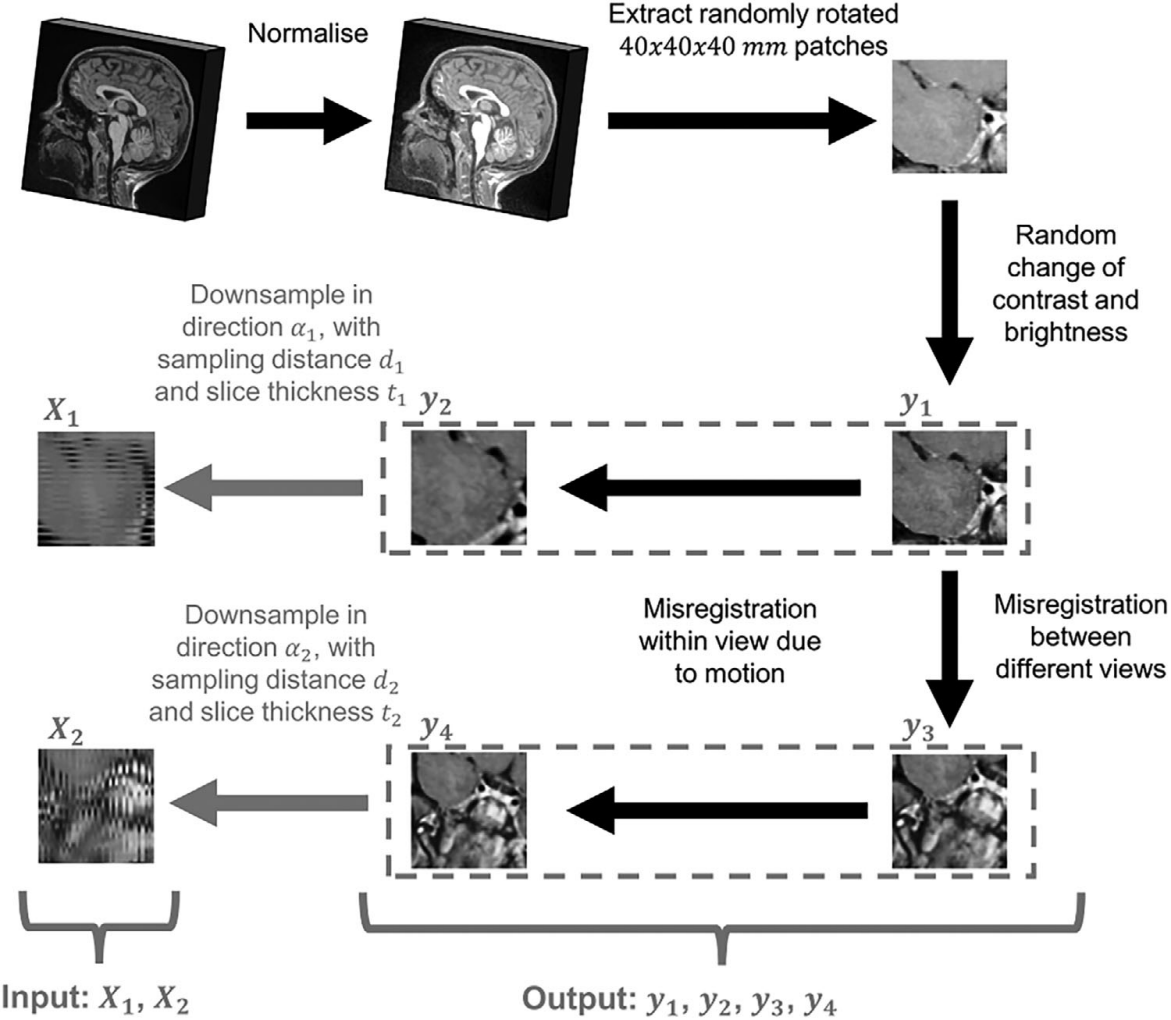
Summary of the downsampling algorithm that generates simulated versions of two perpendicular anisotropic low‐resolution views of the same 40 × 40 × 40 mm patch, $x_{1}$ and $x_{2}$, which show LR motion artifacts, as well as contrast changes and misregistration. The super‐resolution model is trained to take as input $x_{1}$ and $x_{2}$, and predict one of the four rotated high‐resolution versions of the patch, $y_{1}$, $y_{2}$, $y_{3},$ or $y_{4}$. LR, low‐resolution.

To train the mDCSRN model, 256 patches were randomly sampled from each of 80 HR training images and rotated randomly so as to make the model agnostic to orientation. These patches were downsampled as described above, generating a training set ${\left\{X_{ij},y_{ik}\right\}}_{i=1..N,j=1,2,k=1..4}$ where N=20480 is the total number of training patches. A summary of the downsampling process is shown in Figure 1, and a more detailed description of the algorithm used is included in Supplementary 1.

The model, which outputs a set of predictions ${\left\{{\hat{y}}_{i}\right\}}_{i=1..N}$ is trained to minimize the minimum L1 loss between $\hat{y_{i}}$ and the four HR images ${\left\{y_{ik}\right\}}_{k=1..4}$, thus the loss of the network is:

$$Loss=\sum_{i}\min_{k=1..4}\left|\hat{y_{i}}-y_{ik}\right|$$

Further details of the network, its training, and its optimization, are included in Supplementary 2. The mDCSRN network was built using the Python package PyTorch^41^ and trained using the package PyTorch Lightning.^42^

### 2.4 Performance metrics

The performance of both the super‐resolution reconstruction network and the iterative downsampling approach was first validated by measuring the visual similarity between simulated and acquired HR images. This performance was considered in comparison to the performance of aligning the LR images through registration, linearly interpolating them to the desired resolution, normalizing them, and combining them through averaging. This pipeline, which we will refer to as linear interpolation is considered as a fast and simple alternative to super‐resolution models. In addition, we report visual quality metrics of the same interpolation pipeline where we use Lanczos resampling which we refer to as Lanczos interpolation. Datasets 1 and 2 were processed through the same data pipeline used in training the network, generating 256 HR and LR patch pairs per scan in the dataset. In cases where the in‐plane resolution of the scans is smaller than 1mm, the input is first interpolated down to 1mm, and the output is later resampled back to the original in‐plane resolution using linear interpolation.

After the super‐resolution model is applied to the LR images, the accuracy of the network was calculated through its mean peak signal‐to‐noise ratio (PSNR), defined as:

$$\mathrm{PSN}R=\frac{1}{N}\;\sum_{i=1..N}10\log_{10}\frac{max{\left(y_{i}\right)}^{2}}{MSE\left(y_{i},\hat{y_{i}}\right)}$$

$max(y_{i})$ is the maximum intensity found in the HR image, and $MSE(y_{i},\hat{y_{i}})$ is the mean squared error between the HR image and the super‐resolution reconstruction. Although a good metric of signal equality, mean squared error, and by extension PSNR, is not necessarily analogous to visual similarity.^43^ Because of this, the structural similarity index measure (SSIM)^44^ of the HR to the reconstructed scans was also used. Since the SSIM is measured in two dimensions, we calculate the average value over the axial, coronal, and sagittal directions. We, in addition, report the mean squared error (MSE), as well as the normalized root mean squared error (NRMSE). We report the standard deviations of the reported PSNR and SSIM over all patches in the dataset for the model and the two interpolation methods.

In addition, we test the reconstruction methods' visual quality in entire images, with both LR scans downsampled to a through‐plane sampling distance of 7 mm and slice thickness of 5 mm, as this is representative of routinely acquired follow‐up LR scans. HR images are now downsampled as a whole, and the LR images are reconstructed into a HR prediction with each respective method. Some scans contain large volumes of empty space outside of the patient's head, for which the reconstruction's quality is not important, and these areas were masked out in order to get estimates of the visual quality metrics for each method in each scan individually.

For the real‐world data of LR and HR pairs, visual similarity metrics are not useful for determining the success of the reconstructions. Indeed, since the LR scans were acquired with different MRI sequences compared to the HR ones, the reconstructions will also appear as of a different sequence, resulting in large differences in tissue contrast, irrespective of reconstruction choice. We focus instead on the ability of the model to improve the structural quality of the scan so as to better extract relevant structural biomarkers from LR scans, which is the original motivation for this work. This will be measured by comparing auto‐contours generated using the commercial software LimbusAI,^45^ from Dataset 1, on 10 pairs of simulated LR images and acquired HR images, and Dataset 3, with 6 pairs of acquired LR and HR images. For Dataset 1, the LR images were simulated as above. In the case of acquired LR images of Dataset 3, these were registered to each other before being fed to the mDCSRN model or interpolation method. Afterwards, the output of either reconstruction method was registered again, this time to the HR reference, before contouring to avoid orientation issues that were not related to the structural consistency of the images. The LimbusAI model generates contours from T1 MR axial images of the eyes, the hippocampi, the brainstem, and the optic nerves, organ at risk structures that are routinely contoured for brain radiotherapy. The consistency between the contours generated from the simulated images and those from the acquired HR images was evaluated through the mean distance‐to‐agreement (mDTA),^46^ 95% Hausdorff distance (95HD),^47^ and Dice similarity coefficient (DSC). As Limbus AI is not validated for pediatric data, we carefully inspected the segmentations to check their quality before calculating these metrics. The 95% confidence intervals of the mean of the two metrics across the different images of dataset 1 and dataset 3 were reported for the two models. Wilcoxon signed‐rank tests were performed and reported between the performance measures of the mDCSRN model and that of the linear interpolation method. The checklist for AI in Medical Physics^48^ for this work is included as Supplementary 3.

## 3 RESULTS

### 3.1 Model characteristics

We found the optimal parameters of the mDCSRN network to be a growth rate of 8, 8 blocks, and 4 units per block, with 248K parameters in total. The final training regime of the network lasted 213 h on a Linux machine with 4 NVIDIA GeForce RTX 2080 Ti GPUs.

### 3.2 Perceptual quality

**FIGURE 2:**
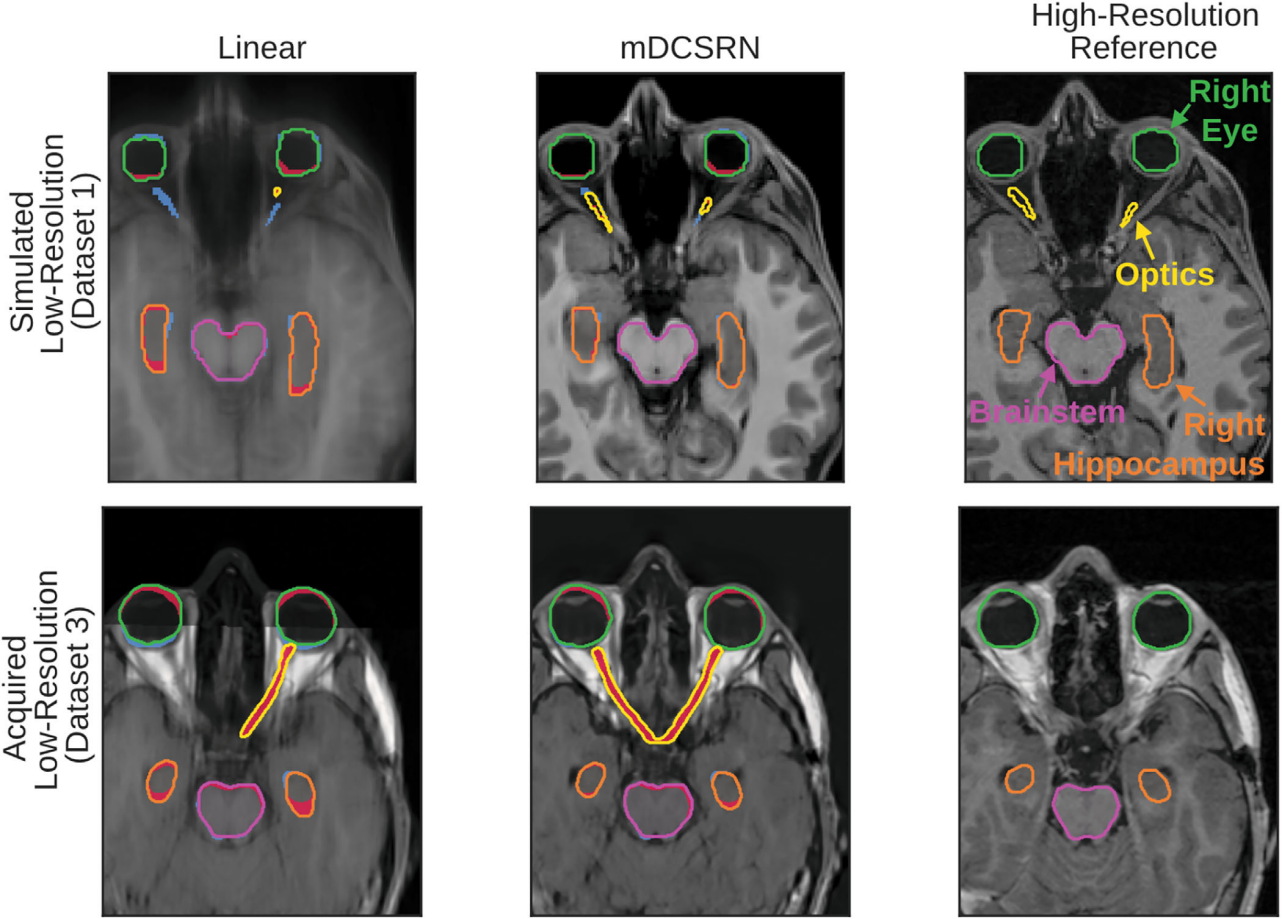
Axial views of example scan reconstructions (Linear interpolation and our model's, mDCSRN, reconstruction), compared to the high‐resolution reference image. The first row is an example from Dataset 1, where LR inputs were simulated, and the second row is an example from Dataset 3, where both the input and reference were acquired MR images. All images are labeled with auto‐segmented structures. In the reconstructions, red masks are overlayed in over‐contoured regions and blue ones in under‐contoured regions, as compared to the HR contour. These examples were taken as the images that had the closest mDTA to the median of its dataset for the mDCSRN model in the contours of the eyes, brainstem, and hippocampi. HR, high‐resolution; LR, low‐resolution; MR, magnetic resonance; mDCSRN, multilevel, densely connected, super‐resolution convolutional neural network.

Visually, the increase of quality in predictions from LR inputs of the mDCSRN compared to the two interpolation methods is clear, with the reconstructions generally better conserving the structural shape of head structures (Figure 2). 3D comparison videos between the reconstructions are included as supplementary material (Supplementary 4).

**FIGURE 3:**
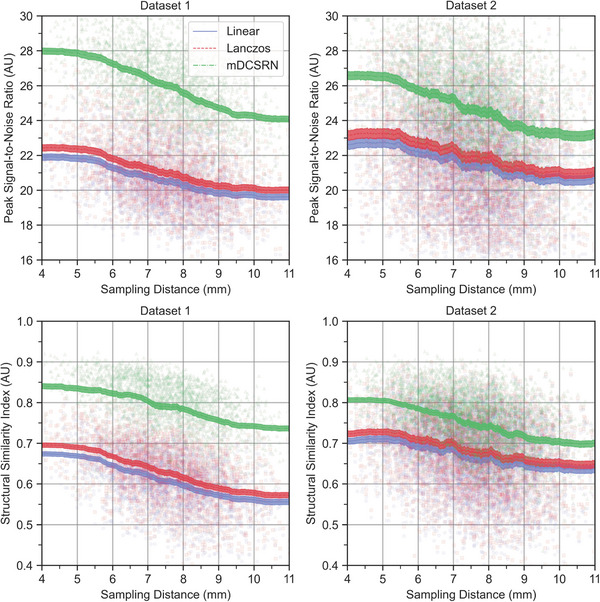
Performance of the deep learning model (mDCSRN) and interpolation in terms of perceptual quality for Datasets 1 and 2, with plots of both the average peak signal‐to‐noise ratio (PSNR) and structural similarity index (averaged over axial, coronal, and sagittal views) as a function of the average sampling distance of the two LR inputs. 95% Confidence Intervals are shaded, and the individual patch performances are shown as dots. LR, low‐resolution; mDCSRN, multilevel, densely connected, super‐resolution convolutional neural network; mDTA, mean distance‐to‐agreement; PSNR, peak signl‐to‐noise ratio.

In terms of visual similarity to the HR acquired images, the super‐resolution neural network outperformed the interpolation methods in Datasets 1 and 2, both in terms of PSNR and SSIM (Figure 3). For the first dataset, the patches generated by the mDCSRN had an average (standard deviation) PSNR of 26.1(2.1), while those reconstructed by the linear interpolation reported one of 20.5(1.9). For the Dataset 2, the two models had a PSNR of 24.4(2.6) and 21.4(2.8) respectively. For the first dataset, the mDCSRN method reports an in‐plane SSIM (along the down‐sampled direction) of 0.80(0.07) and a through‐plane (along the other two directions) SSIM of 0.79(0.07), while the linear interpolation method reports 0.62(0.07) and 0.62(0.07), respectively. For Dataset 2, the two SSIM are 0.75(0.08) and 0.72(0.08) for the mDCSRN, while they are 0.68(0.10) and 0.63(0.09) for the linear interpolation.

Comparing the performance for different sampling distances and slice thicknesses, the network's performance deteriorates when the input scans have lower through‐plane resolution. For the two datasets, generated images from input scans with sampling distance higher than 7.5 mm have a PSNR of 23.8(2.4), an in‐plane SSIM of 0.73(0.08), and a through‐plane SSIM of 0.71(0.08). Generated images from inputs where one scan had a resolution under 7.5 mm, the PSNR was 25.2(2.5), the in‐plane SSIM was 0.77(0.07), and the through‐plane SSIM was 0.75(0.08). For scans for which both inputs had resolutions under 7.5  mm, the PSNR was 26.1(2.4), the in‐plane SSIM was 0.80(0.07) and the through‐plane SSIM was 0.78(0.07).

The whole‐image visual quality metrics, including the MSE and NRMSE are presented in Table 2, and their related boxplots are included in Supplementary 5.

**TABLE 2:** Performance of the linear interpolation method (Linear), Lanczos interpolation method (Lanczos), and dense convolutional neural network (mDCSRN) in the full‐image visual task in terms of visual quality metrics.

	Dataset 1			Dataset 2		
Reconstruction method	Linear	Lanczos	mDCSRN	Linear	Lanczos	mDCSRN
Performance metric						
Mean squared error (MSE)	208.1	189.2	**99.0**	5382.4	4780.3	**2669.2**
Normalized root mean square error (NRMSE)	0.092	0.087	**0.062**	0.094	0.088	**0.069**
Peak signal‐to‐noise ratio (PSNR)	21.1	21.5	**24.4**	21.3	21.9	**24.1**
Structural similarity index (SSIM)						
Axial	0.59	0.61	**0.71**	0.67	0.68	**0.73**
Coronal	0.59	0.61	**0.72**	0.69	0.70	**0.75**
Sagittal	0.57	0.59	**0.70**	0.67	0.68	**0.73**
Overall	0.58	0.60	**0.71**	0.67	0.69	**0.74**

*Note*: The average 2D structural similarity index over the axial, coronal, and sagittal slices is presented, as well as the overall average over the three orientations. The best performing method according to each metric is bolded.

Abbreviation: mDCSRN, multilevel, densely connected, super‐resolution convolutional neural network.

### 3.3 Segmentation task

**FIGURE 4:**
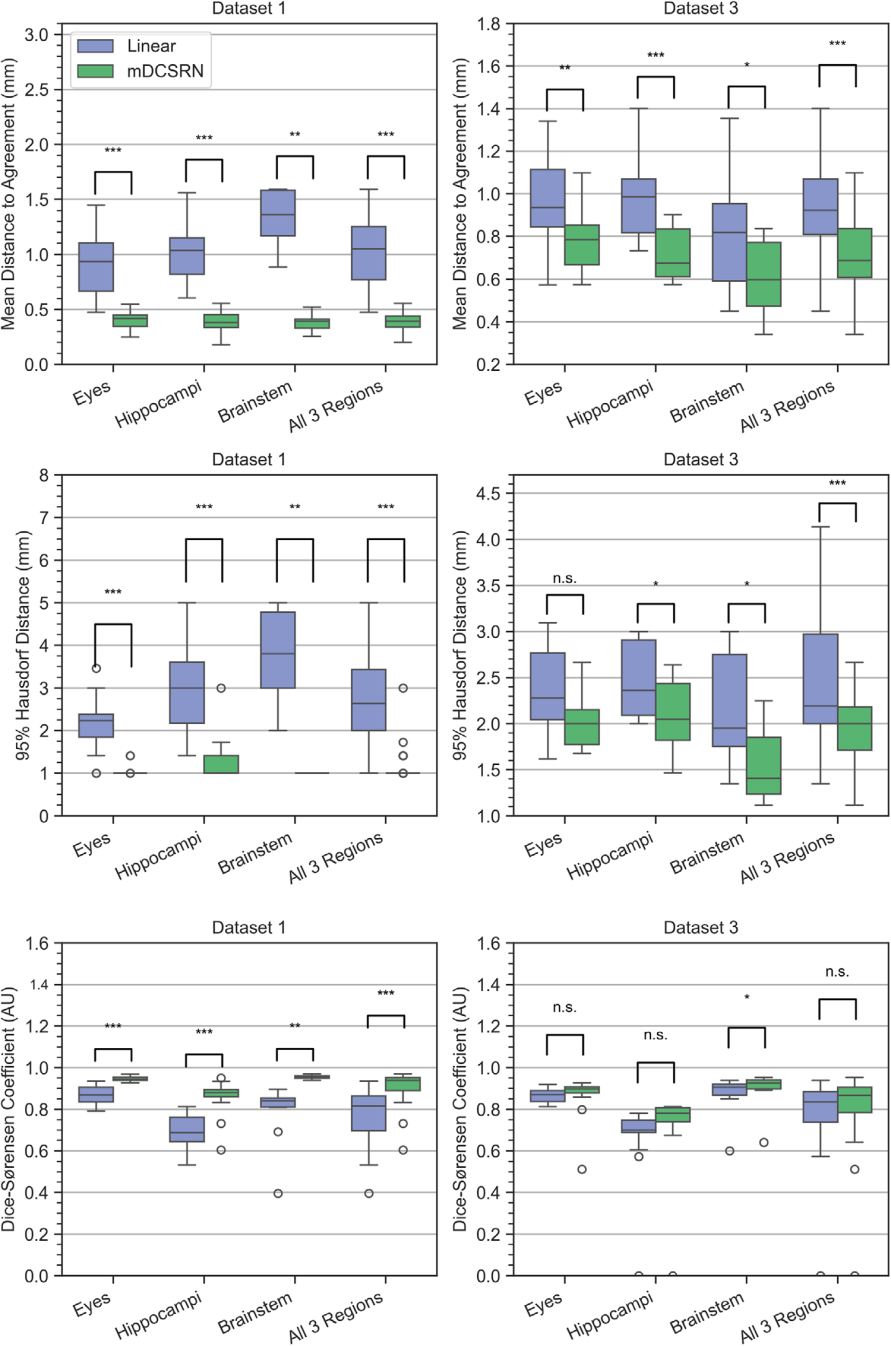
Performance of the dense convolutional neural network (mDCSRN) and interpolation (Linear) in terms of structural consistency of auto‐segmented structures with the auto‐contoured HR images for Datasets 1 and 3. The first row shows the mDTA the second shows the 95HD, and the third shows the DSC of the contours. Wilcoxon paired significance tests were carried out to compare the two models and shown as asterisks (with one asterisk meaning *p* < 0.05, two *p* < 0.01, and three *p* < 0.001, n.s. stands for non‐significant). 95HD, Hausdorff distanc; DSC, dice similarity coefficient; mDCSRN, multilevel, densely connected, super‐resolution convolutional neural network; mDTA, mean distance‐to‐agreement.

Similar to perceptual quality, the contours generated from the output of the mDCSRN conserved better the shape and volume of the HR contours compared to both interpolation methods (Figure 2). Images, with and without contours, from the patients of both datasets with the worst and best mDTA, as well as the patients from Figure 2 without overlaid contours are included in Supplementary 6. Axial, coronal, and sagittal slices of each of these images are also included in Supplementary 6. Quantitatively, contours generated on the scans predicted by the mDCSRN were more consistent compared to those created from interpolated scans (Figure 4). The contours from the mDCSRN prediction had an average (95% CI) mDTA of 0.7(0.4−1.0)mm in Dataset 1 and 0.8(0.7−0.9)mm in Dataset 3, and an average 95HD of 2.4(1.3−3.5)mm for Dataset 1 and 2.6(1.9−3.2)mm for Dataset 3. The contours on linearly interpolated scans gave an average mDTA of 6.5(3.6−9.5)mm in Dataset 1 and 1.2(1.0−1.3)mm in Dataset 3, and an average 95HD of 11.1(8.2−14.1)mm for Dataset 1 and 3.5(2.9−4.1)mm for Dataset 3.

The structure that was least consistent with respect to that of the HR scans for both models was the optic nerves, with the mDCSRN showing an average contour mDTA of 2.2(0.4−4.0)mm in Dataset 1 and 1.2(0.7−1.7)mm in Dataset 3, and a 95HD of 8.7(2.5−14.9)mm in Dataset 1 and 5.6(2.2−9.0)mm in Dataset 3. The linear interpolation still had a worse performance, with an average mDTA of 33.3(16.1−50.5)mm in Dataset 1 and 2.2(1.1−3.3)mm in Dataset 3. The 95HD for interpolation was 50.3(35.0−65.6)mm for Dataset 1 and 8.7(5.1−12.3)mm for Dataset 3. When excluding the optics the average contour mDTA, for mDCSRN, was 0.40(0.37−0.44)mm for Dataset 1 and 0.71(0.66−0.78)mm for Dataset 3 and, for the Linear method, 1.18(0.90−1.46)mm for Dataset 1 and 0.95(0.86−1.04)mm for Dataset 3, as seen in Figure 4.

The average DSC of the mDCSRN reconstruction was higher than that of the Linear ones in both datasets, although these differences were small. Average values for the mDTA, 95%HD, and DSC for each reconstruction method, dataset, and structure are presented in Table 3.

**TABLE 3:** Performance of the linear interpolation method (Linear) and dense convolutional neural network (mDCSRN) in the full‐image segmentation task in terms of the structural similarity of segmentations compared to those done on a reference high‐resolution image.

	Dataset 1		Dataset 3	
Reconstruction method	Linear	mDCSRN	Linear	mDCSRN
Performance metric				
Mean distance‐to‐agreement (mDTA, in mm)				
Brainstem	2.04	**0.38**	0.83	**0.61**
Eyes	0.91	**0.40**	0.97	**0.78**
Optics	33.27	**2.20**	2.25	**1.24**
Hippocampi	1.03	**0.41**	0.99	**0.71**
95% Hausdorff distance (mDTA, in mm)				
Brainstem	6.31	**1.00**	2.16	**1.56**
Eyes	2.14	**1.06**	2.48	**2.08**
Optics	50.32	**8.72**	8.68	**5.64**
Hippocampi	2.97	**1.24**	2.72	**2.07**
Dice similarity coefficient (DSC)				
Brainstem	0.79	**0.95**	0.90	**0.93**
Eyes	0.87	**0.95**	0.87	**0.90**
Optics	0.07	**0.51**	0.39	**0.46**
Hippocampi	0.69	**0.86**	0.71	**0.77**

*Note*: Each metric is presented in each of the segmented regions separately. The best performing method according to each metric is bolded.

Abbreviation: mDCSRN, multilevel, densely connected, super‐resolution convolutional neural network; mDTA, mean distance‐to‐agreement.

## 4 DISCUSSION

In this paper, we have built a multiscan MRI reconstruction method that uses a dense convolutional neural network. The images produced by the model were more visually accurate, but most importantly were better for the extraction of structural biomarkers, with mDTA and 95HD values that were below those of interpolation, and which are smaller than what would be normally expected due to interobserver variability.^49^ The improvements in the DSC were smaller. Previous work has criticized the use of this metric when evaluating the quality of segmentations in radiotherapy, due to its limited sensitivity to boundary errors.^50^, ^51^ However, volumetric and distance‐based measures can be complementary in assessing the structural quality of segmentations.^50^

Oktay et al.^29^ and Jurek et al.^30^ previously explored the use of convolutional neural networks to improve the potential of super‐resolution multislice MR reconstruction, in the heart and brain, respectively. However, their work, which tested both visual and structural consistency between outputs and targets, was tested only in images for which the LR inputs were simulated. As shown in this work, differences between the downsampling pipeline and real acquired LR images are a great source of bias for the model. Given the modest difference in performance between the datasets 1 and 2, and the larger gap between datasets 1 and 3, this seems to be a bigger source of model error than generalizability problems across different scanners, contrasts, and morphologies, which have also been raised as problematic in the use of deep learning for medical physics problems.^52^ This is the first work testing a deep learning solution in LR fusion focusing on brain and optic structures related to long‐term effects of radiotherapy in children. The network's performance was considered acceptable for the structural analysis of the eyes, hippocampi, and brainstem. Although the contouring of the optic nerves was more difficult due to their thin shape, the model still outperformed interpolation for this structure. Due to differences in sequences and tissue contrast between the LR and HR pairs, we were not able to assess the visual quality of reconstructions in real‐world images, focusing instead on their structural quality. Future work could use deep learning methods to transform outputs to a target sequence,^53^ permitting validation of visual similarity when low and HR sequences differ.

We implemented a range of data augmentation operations within our downsampling and training pipeline to make our model more appropriate for realistic follow‐up images, as well as more robust to distributional shift due to changes in scanners or medical context. Researchers have developed a range of data augmentation methods to improve the generalizability of statistical medical imaging models,^54^ like geometric transforms, cropping, contrast change, blurring, and a combination of them. We applied these to the extent that we deemed possible for our problem, but further, more complicated data transformation approaches, such as deformable augmentation,^54^ could be explored. Researchers have used probabilistic generative model segmented label maps to generate synthetic images with a wide array of contrast, improving the robustness of downstream models.^55^, ^56^ We however considered that it was important to retain the high‐quality realistic targets in our dataset, and thus chose simpler methods that would retain the quality of our images. Deep learning generative models like generative adversarial networks (GANs)^57^ or diffusion models^58^ could potentially be used to generate a training dataset with both the diversity in features and contrasts and the realism required for super‐resolution applications.

Further data augmentation to create a more realistic downsampling pipeline could reduce further the ``simulated‐acquired'' performance gap, as well as expanding the variation in the training dataset. Although we used Gaussian blurring to simulate different slice thicknesses in the LR images, uniform blurring could also be explored, as well as using a different range for the Gaussian kernel. In terms of methodological advances, further neural network architectures could be explored to improve the performance of the model. Although GANs have often been used to improve the visual ``realism'' of images in super resolution applications,^27^, ^36^, ^59^, ^60^, ^61^ we trained a conventional neural network, as adversarial training did not seem to improve our model in initial sensitivity studies. Additionally, the use of transformers in computer vision tasks is being explored,^62^ including in super‐resolution tasks.^63^ The recent success of diffusion models in generative visual tasks^64^ has encouraged researchers to explore its use also in super‐resolution problems.^65^ In order to improve the trustworthiness and interpretability of the model outputs, Bayesian methods could be applied to provide confidence intervals on certain voxel regions of the output, warning the users of situations in which the model might be unreliable.^66^

A limitation of this work is the lack of benchmarking of our model to constrained reconstruction methods. This was partly due to the fact that the ``raw'' MRI data needed for this approach were not available in this study. Moreover, since the motivation for this work was the reconstruction of HR images for retrospective studies using routinely acquired data, for which these files would almost never be available, we considered appropriate to omit comparisons against these methods in our work. A comparison between this model or other deep learning approaches and constrained reconstruction methods would; however, still be of interest in further work. Similarly, we did not compare our method to single‐image deep learning reconstruction methods. Usually more than one set of low‐resolution slices is available in retrospective follow‐up studies, as more than one direction is needed for diagnosis. Moreover, slice distances studied in this paper exceeded what is usually studied in single‐image isotropic super‐resolution studies.^27^, ^60^, ^67^ This motivated our decision not to evaluate a single‐image super‐resolution algorithm.

Although we validated the model on real‐world data, it only constitutes one example of a patient at follow‐up. Further validation work needs to be carried out with larger diversity of patient morphology, scanner type, field of view, and structures of interest. In addition, in order to better understand the adequacy of deep learning for the creation of retrospective cohorts for temporal structural analysis, it would also be beneficial to study the consistency between the morphological trends extracted in model outputs and those extracted from HR reference images. The sample size of this study could not accommodate for subgroup analysis. Further study across different patient characteristics, such as performance differences between healthy individuals and patients with cancer, before and after treatment, would be of interest.

## 5 CONCLUSION

We investigate the potential of deep learning in the reconstruction of high‐resolution MR scans from routinely acquired thick sliced images in pediatric head and neck cancer patients. We used a dense convolutional neural network, trained on simulated data from a set of HR scans. The resulting model outperformed interpolation both in terms of perceptual and structural quality, both in simulated and real LR MR scans. Contours generated on our model output showed good consistency with those derived from HR scans, indicating that our model output is suitable for structural analysis.

This work has potential applications in the analysis of structural biomarkers in retrospective studies, particularly for pediatric cancer survivors, where available clinical scans often lack the necessary resolution for such measurements. Deep learning could unlock routinely acquired pediatric data for further analysis to help us to explore dose‐response relationships in radiotherapy, providing clinical evidence for biological effectiveness and long‐term side effects to improve future treatments.

## CONFLICT OF INTEREST STATEMENT

The authors have no relevant conflicts of interest to disclose.

## Supporting information



Supporting Information

## Data Availability

The datasets used in this work are not readily available because ethical permission was not granted for general publication of the dataset. Further information can be found in https://abcdstudy.org and https://cbtn.org/ and by contacting Dr. Angela Davey (angela.davey@manchester.ac.uk).
