# Supplementary material for: A neural network to create super‐resolution MR from multiple 2D brain scans of pediatric patients
Source: Med Phys. 2024 Dec 10;52(3):1693–705. doi: 10.1002/mp.17563 (PMC11880662; doi:10.1002/mp.17563)
Supplement: Supplementary file 1 — Supporting Information [file MP-52-1693-s001.zip › Supplementary 1.docx]

Supplementary 1 Table 1: Data pipeline algorithm for the creation of the training and optimization datasets.

**BuildTrainingData:** Generates a dataset to train the super-resolution network with.

Input:

$\{H_{m}{\}}_{m=1..M}$: Set of high-resolution images of a patient’s head.

$s$: Number of patches to sample per image.

Output:

$\{X_{ij},y_{ik}{\}}_{i=1..N;j=1,2; k=1..4}$: Set of pairs of high-resolution and corresponding low-resolution 3D images.

Initialize output $O = \{\}$.

**for each** $H\in\{H_{m}{\}}_{m=1..M}$ **do**

Normalize the values of $H$ to have a mean of 0 and standard deviation of 1.

**for each** $s\in\left[ 1,2,..,S \right]$ **do**

Rotate $y_{1}$ by a random angle (360 °) and direction.

Sample a 40 x 40 x 40 patch $y_{1}$ from $H$.

Change the contrast and brightness (up to factors of 0.4, using torchvision) of $y_{1}.$

Generate a copy $y_{2}$ of $y_{1}$ and rotate it with random direction and angle of $\theta\in U\left( -5^{\circ},5^{\circ} \right)$.

Generate two downsampling directions $\alpha_{1}$ and $\alpha_{2}$ which are perpendicular from each other.

**For each** $y_{i}\in\{y_{1},y_{2}\}$ **do**

Generate a copy $y_{i+2}$ of $y_{i}$ and rotate it with random direction and angle of $\psi\in U\left( -5^{\circ},5^{\circ} \right)$.

Generate copies $X_{i}$ and $X_{i+2}$ of $y_{i}$ and $y_{i+2}$ respectively.

Apply isotropic gaussian blur of equal random kernel size and standard deviation to $X_{i}$ and $X_{i+2}$.

Apply random noise of equal random intensity to $X_{i}$ and $X_{i+2}$.

Downsample $X_{i}$ and $X_{i+2}$ in the direction $\alpha_{i}$, with random sampling distance
 $d_{i}= Uniform(3 mm, 8 mm)$ and slice thickness $t_{i}=Uniform\left( d_{i},d_{i}+4 mm \right)$.

Substitute the even slices of $X_{i}$ by those of $X_{i+2}$.

With 10% probability, partly or fully obscure either $X_{1}$ or $X_{2}$.

Append $\{X_{1},X_{2},y_{1},y_{2},y_{3},y_{4}\}$ to $O$.
