# Supplementary material for: A neural network to create super‐resolution MR from multiple 2D brain scans of pediatric patients
Source: Med Phys. 2024 Dec 10;52(3):1693–705. doi: 10.1002/mp.17563 (PMC11880662; doi:10.1002/mp.17563)
Supplement: Supplementary file 1 — Supporting Information [file MP-52-1693-s001.zip › Supplementary 2.docx]

Supplementary 2: Details of the neural network and the training and optimization process.

The model used was a densely connected neural network, as presented in Chen et al. (DOI: <https://doi.org/10.48550/arXiv.1803.01417>). Detailed details of the architecture of the network are explained in the original paper and summarized below.


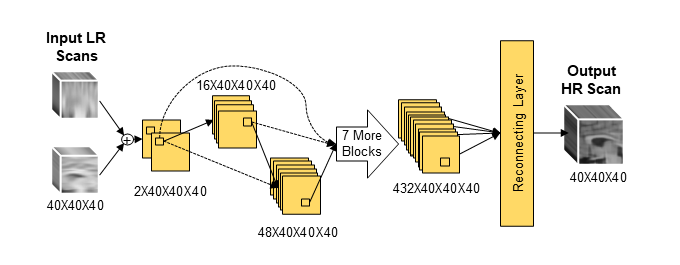


The network is characterized by the following hyperparameters: the number of blocks in the network U, the number of units in each block U and the growth rate G. The network takes as input the two interpolated low-resolution scans as a 4D tensor of size 2x40x40x40 and uses a 3D convolutional layer of kernel size 3 to output a 2Gx40x40x40 tensor. This tensor is then passed to the main component of the network: a sequence of blocks composed of units defined as a 3D Batch Norm layer, a Leaky ReLU activation function, and a convolutional layer of kernel size 3 which outputs a tensor of size Gx40x40x40. The input of each unit is the concatenation of the previous outputs of all units before it within the block, and the input of each block is the concatenation of the previous blocks in the network. Finally, a reconnecting convolutional layer of kernel size 1 takes the outputs of all blocks, concatenated as a tensor of size ((B+1)U + 2B + 2)Gx40x40 x40, and reconnects them into the final output of size 1x40x40x40.

The network was trained for 60 epochs, with a learning rate of 0.0001 using the learning algorithm AdamW. The hyperparameters were chosen through grid selection, for which the parameter set with the highest structural similarity index (averaged over the axial, sagittal and coronal views) in the holdout tuning set of 10 HR held out images (from which 25,600 patches were extracted) was chosen. The growth rates G explored were {8,10,12}, the number of blocks explored were {6,8,10} and the number of units was {3,4,5}.
