# Supplementary material for: A neural network to create super‐resolution MR from multiple 2D brain scans of pediatric patients
Source: Med Phys. 2024 Dec 10;52(3):1693–705. doi: 10.1002/mp.17563 (PMC11880662; doi:10.1002/mp.17563)
Supplement: Supplementary file 1 — Supporting Information [file MP-52-1693-s001.zip › Supplementary 3.docx]

Supplementary 3 Table 1: Checklist for AI in Medical Physics.

| Indicate whether each section clearly summarizes or describes: | Checkboxes | | | Page Number |
| --- | --- | --- | --- | --- |
| 1. Abstract | Yes | No | N/A |  |
| a. Purpose, rationale, novelty or significance | X |  |  | 1 |
| b. AI/ML methods and data type, dataset partitioning into training, validation (tuning), and test sets (include  numbers used in training, validation, and test sets) | X |  |  | 1 |
| c. Main results, including statistical analyses | X |  |  | 1 |
| 2. Introduction | | | | |
| a. Purpose and justification of using AI/ML algorithm approach | X |  |  | 2-3 |
| b. Contribution(s) of AI/ML to medical physics application | X |  |  | 2-3 |
| c. Stage of development (e.g., pilot study, mature study) | X |  |  | 3 |
| 3. Materials | | | | |
| a. Dataset characteristics including sample size and clinical acquisition sites | X |  |  | 3-4 |
| b. Device(s) used for data acquisition (e.g., scanner makes), start-end dates of acquisition (or equivalent means with biotechnology generated data), and any data harmonization, augmentation, and enrichment strategies, or pre-processing are clearly described | X |  |  | 3-4 |
| c. For imaging data: image or data acquisition modality, acquisition protocol, or parameter ranges are detailed | X |  |  | Tab 1 |
| d. For patient data: method to obtain the sample, representativeness of the population for the purpose of the study, IRB approval (or equivalent), and relevant patient demographics plus clinical variables such as prevalence(s) of disease(s) or lesion characteristics |  |  | X |  |
| e. For phantom data: Type of phantom and method for generating phantom data |  |  | X |  |
| f. Data composition appropriateness for AI/ML application | X |  |  | 4 |
| g. Description of the “ground truth,” that is, the reference standard, including the annotation process, level of subjectivity, and uncertainty | X |  |  | 5-6 |
| h. Data partitioning into training, validation (tuning), and test sets including any criteria to mitigate bias and justification of sample sizes | X |  |  | 3-4 |
| i. Final validation using public dataset or study dataset to be shared/made publicly available (desirable but not required) |  | X |  |  |
| 4.1 Methods: Machine learning algorithm | | | | |
| a. Methodology in sufficient detail to allow replication, including model architecture, hyperparameters, inputs, dimensionality of the input (e.g., 2D or 3D images), pre-processing, output type and definition, and discretization/binning, if any | X |  |  | 5, Supp 2 |
| b. Training/optimization method including loss function, regularization approach, data imbalance mitigation process (if needed), measures to minimize overfitting and bias, and ablation studies, if any | X |  |  | 5 |
| c. AI/ML software code to be shared/made publicly available (desirable but not required). |  | X |  |  |
| 4.2 Methods: Performance and statistics | | | | |
| a. Performance metric(s) including any postprocessing (such as scoring criteria, decision threshold, binning) of the AI/ML output. | X |  |  | 5-6 |
| b. Method(s) to estimate the uncertainty (such as 95% confidence intervals) of the performance metric(s). | X |  |  | 6 |
| c. Significance of the obtained results compared to the null hypothesis (if applicable) or compared to a suitable benchmark metric. |  |  | X |  |
| d. Subgroup analyses for important subgroups (e.g., by age, lesion size). |  |  |  |  |
| e. Demonstrative results for the training, validation (tuning), and test sets. | X |  |  | 6-7 |
| 5. Discussion | | | | |
| a. Conclusions as supported by the results. | X |  |  | 7-9 |
| b. Limitations of the study. | X |  |  | 8 |
| c. Discussion/summary of innovation (algorithm or application), significance (clinical or scientific), and/or contributions to the field of medical physics. | X |  |  | 7-8 |
