# Supplementary material for: A neural network to create super‐resolution MR from multiple 2D brain scans of pediatric patients
Source: Med Phys. 2024 Dec 10;52(3):1693–705. doi: 10.1002/mp.17563 (PMC11880662; doi:10.1002/mp.17563)

Supplementary 5 Figure 1: Axial views (without contours) of example scan reconstructions (Linear interpolation and our model’s, mDCSRN, reconstruction), compared to the high-resolution reference image. The first row is an example from Dataset 1, where low-resolution inputs were simulated, and the second row is an example from Dataset 3, where both the input and reference were acquired MR images. These examples were taken as the images that had the closest mean distance-to-agreement to the median of its dataset for the mDCSRN model in the contours of the eyes, brainstem and hippocampi.


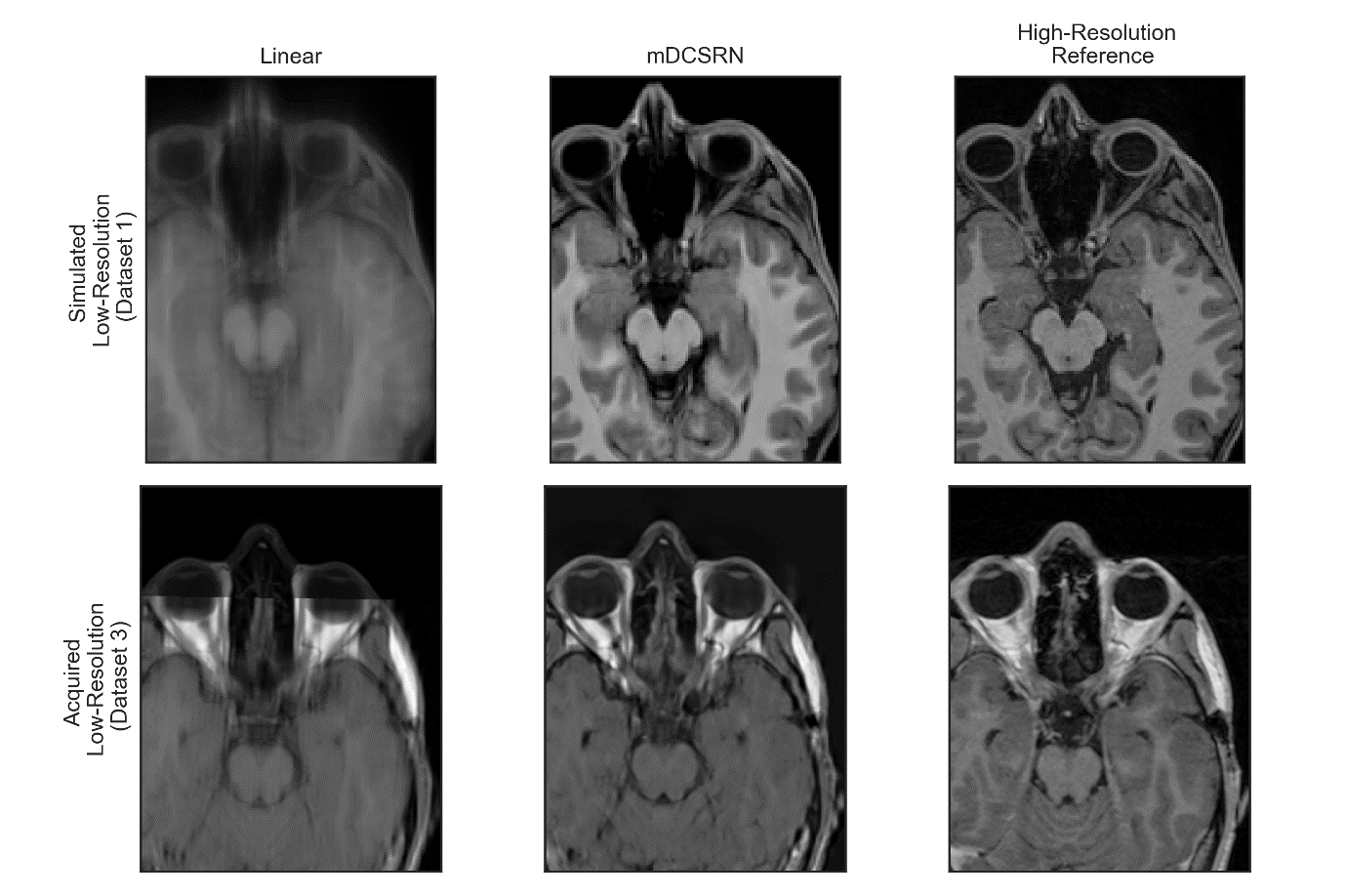


Supplementary 5 Figure 2: Axial views of example scan reconstructions (Linear interpolation and our model’s, mDCSRN, reconstruction), compared to the high-resolution reference image. The first row is an example from Dataset 1, where low-resolution inputs were simulated, and the second row is an example from Dataset 3, where both the input and reference were acquired MR images. All images are labelled with auto-segmented structures (eyes in green, optics in yellow, brainstem in pink and the hippocampi in orange). In the reconstructions, red masks are overlayed in over-contoured regions and blue ones in under-contoured regions, as compared to the high-resolution contour. These examples were taken as the images that had the worst mean distance-to-agreement for the mDCSRN model in the contours of the eyes, brainstem and hippocampi.


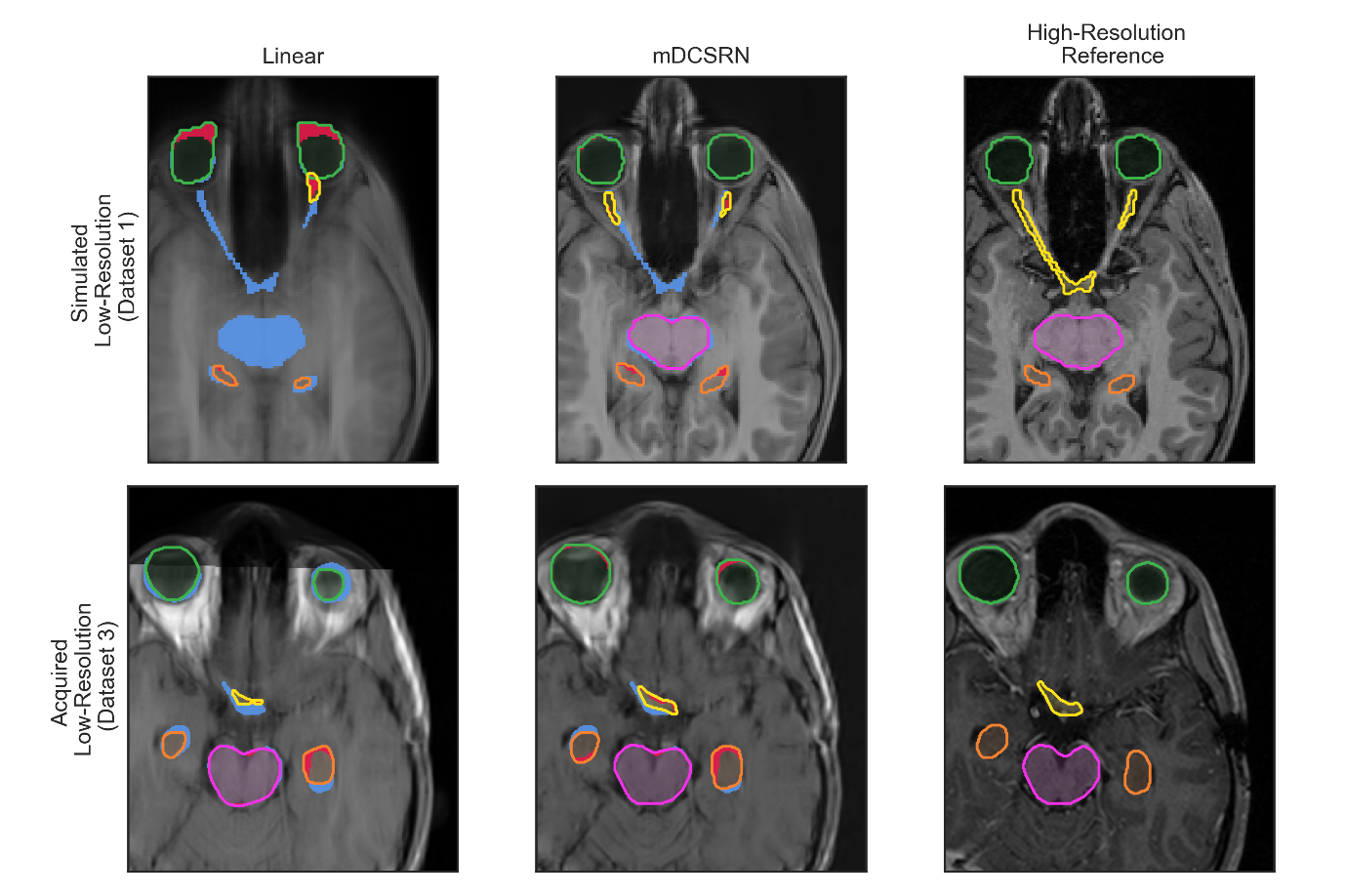


Supplementary 5 Figure 3: Axial views (without contours) of example scan reconstructions (Linear interpolation and our model’s, mDCSRN, reconstruction), compared to the high-resolution reference image. The first row is an example from Dataset 1, where low-resolution inputs were simulated, and the second row is an example from Dataset 3, where both the input and reference were acquired MR images. These examples were taken as the images that had the worst mean distance-to-agreement for the mDCSRN model in the contours of the eyes, brainstem and hippocampi.


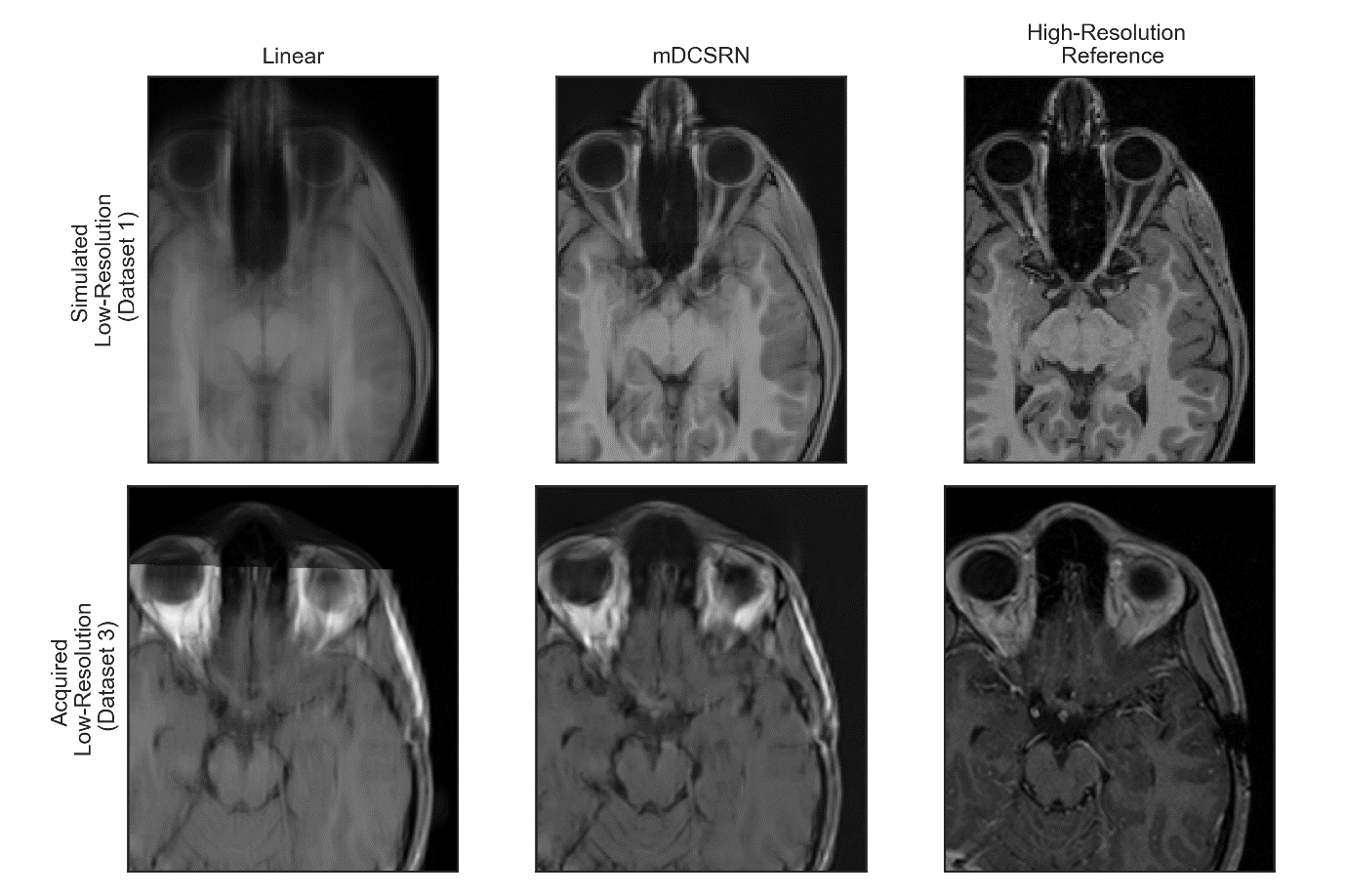


Supplementary 5 Figure 4: Axial views of example scan reconstructions (Linear interpolation and our model’s, mDCSRN, reconstruction), compared to the high-resolution reference image. The first row is an example from Dataset 1, where low-resolution inputs were simulated, and the second row is an example from Dataset 3, where both the input and reference were acquired MR images. All images are labelled with auto-segmented structures (eyes in green, optics in yellow, brainstem in pink and the hippocampi in orange). In the reconstructions, red masks are overlayed in over-contoured regions and blue ones in under-contoured regions, as compared to the high-resolution contour. These examples were taken as the images that had the best mean distance-to-agreement for the mDCSRN model in the contours of the eyes, brainstem and hippocampi.


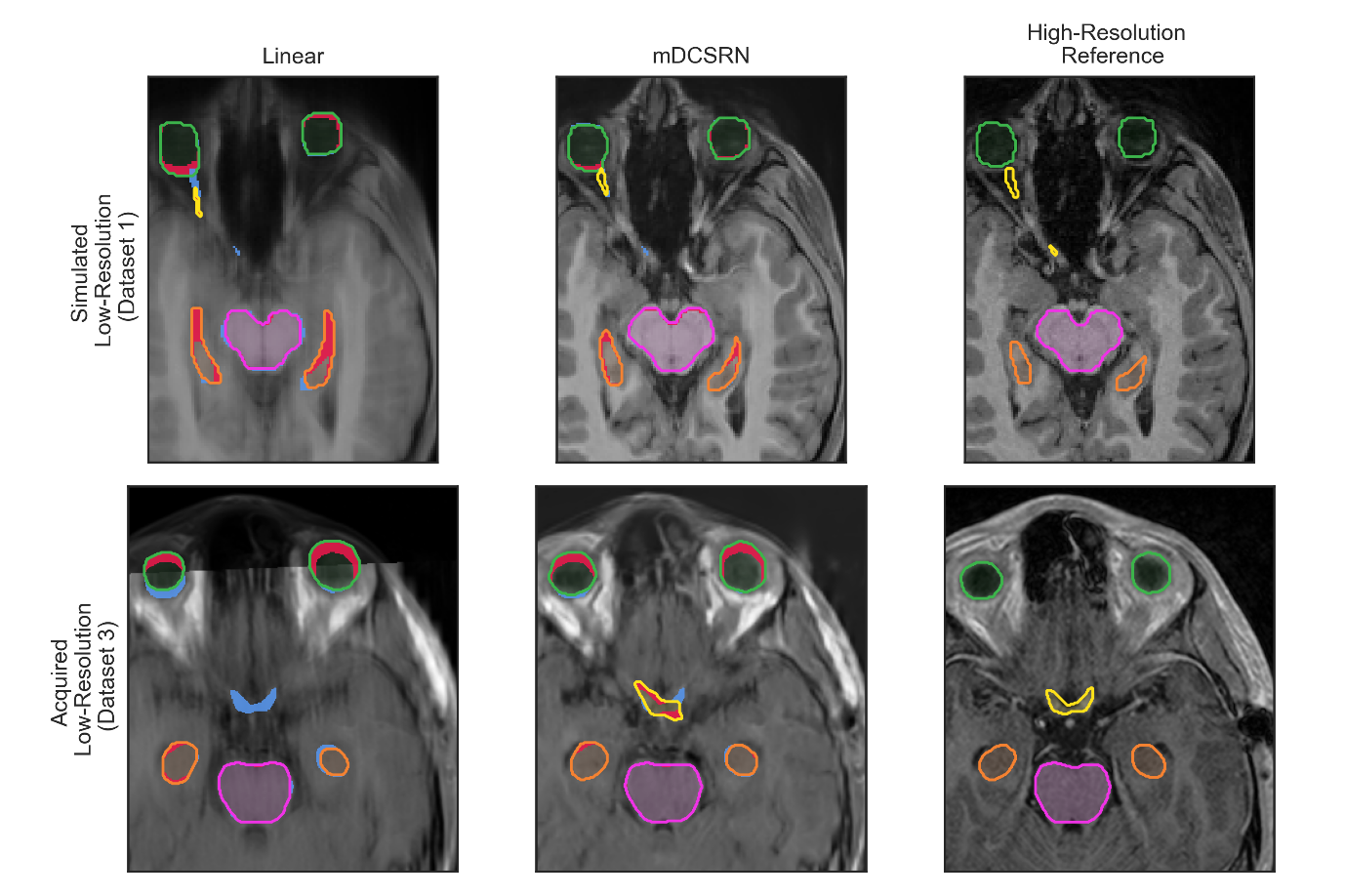


Supplementary 5 Figure 5: Axial views (without contours) of example scan reconstructions (Linear interpolation and our model’s, mDCSRN, reconstruction), compared to the high-resolution reference image. The first row is an example from Dataset 1, where low-resolution inputs were simulated, and the second row is an example from Dataset 3, where both the input and reference were acquired MR images. These examples were taken as the images that had the best mean distance-to-agreement for the mDCSRN model in the contours of the eyes, brainstem and hippocampi.


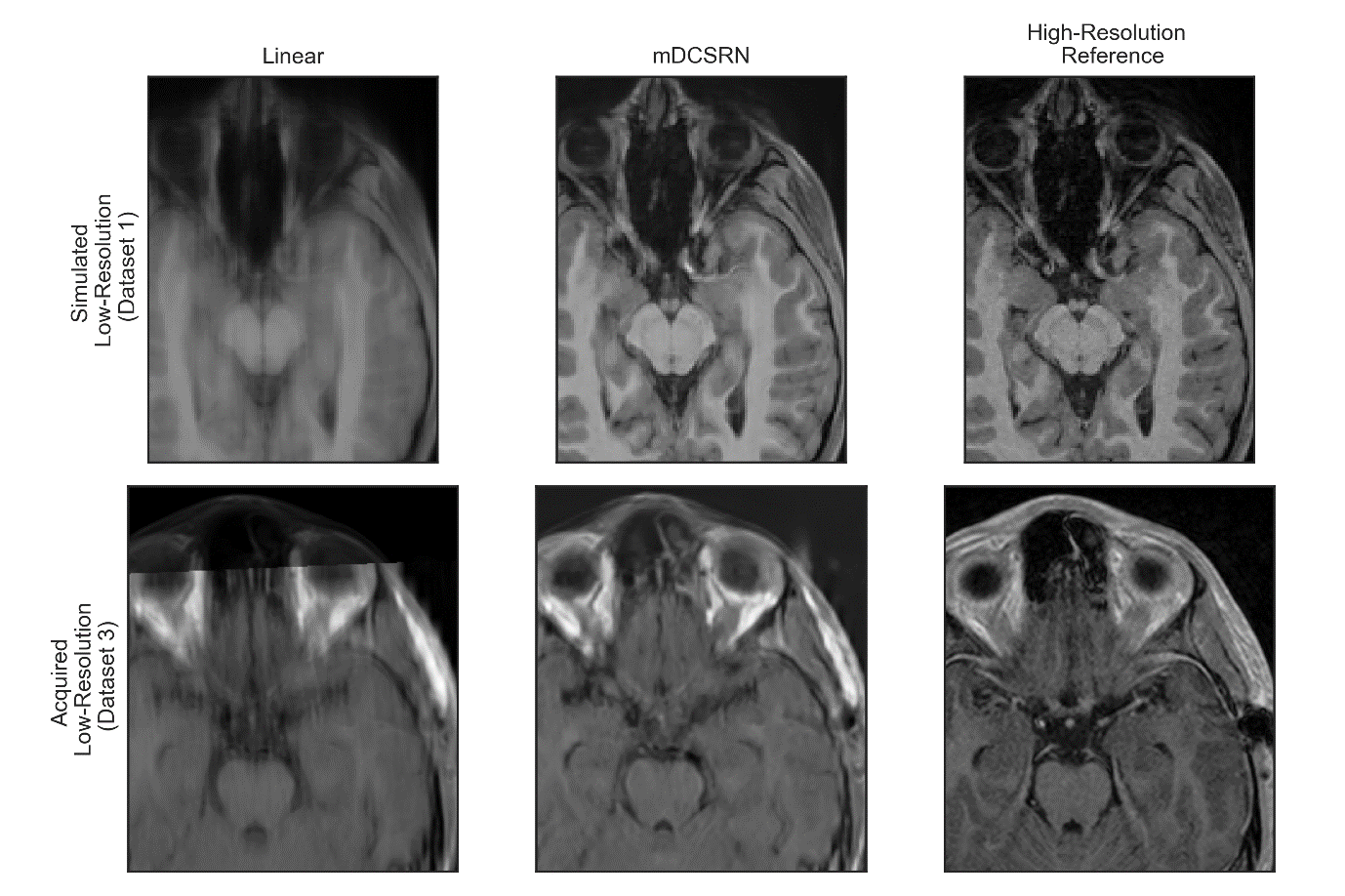


Supplementary 5 Figure 6: Coronal views (without contours) of example scan reconstructions (Linear interpolation and our model’s, mDCSRN, reconstruction), compared to the high-resolution reference image. The first row is an example from Dataset 1, where low-resolution inputs were simulated, and the second row is an example from Dataset 3, where both the input and reference were acquired MR images. These examples were taken as the images that had the closest mean distance-to-agreement to the median of its dataset for the mDCSRN model in the contours of the eyes, brainstem and hippocampi.


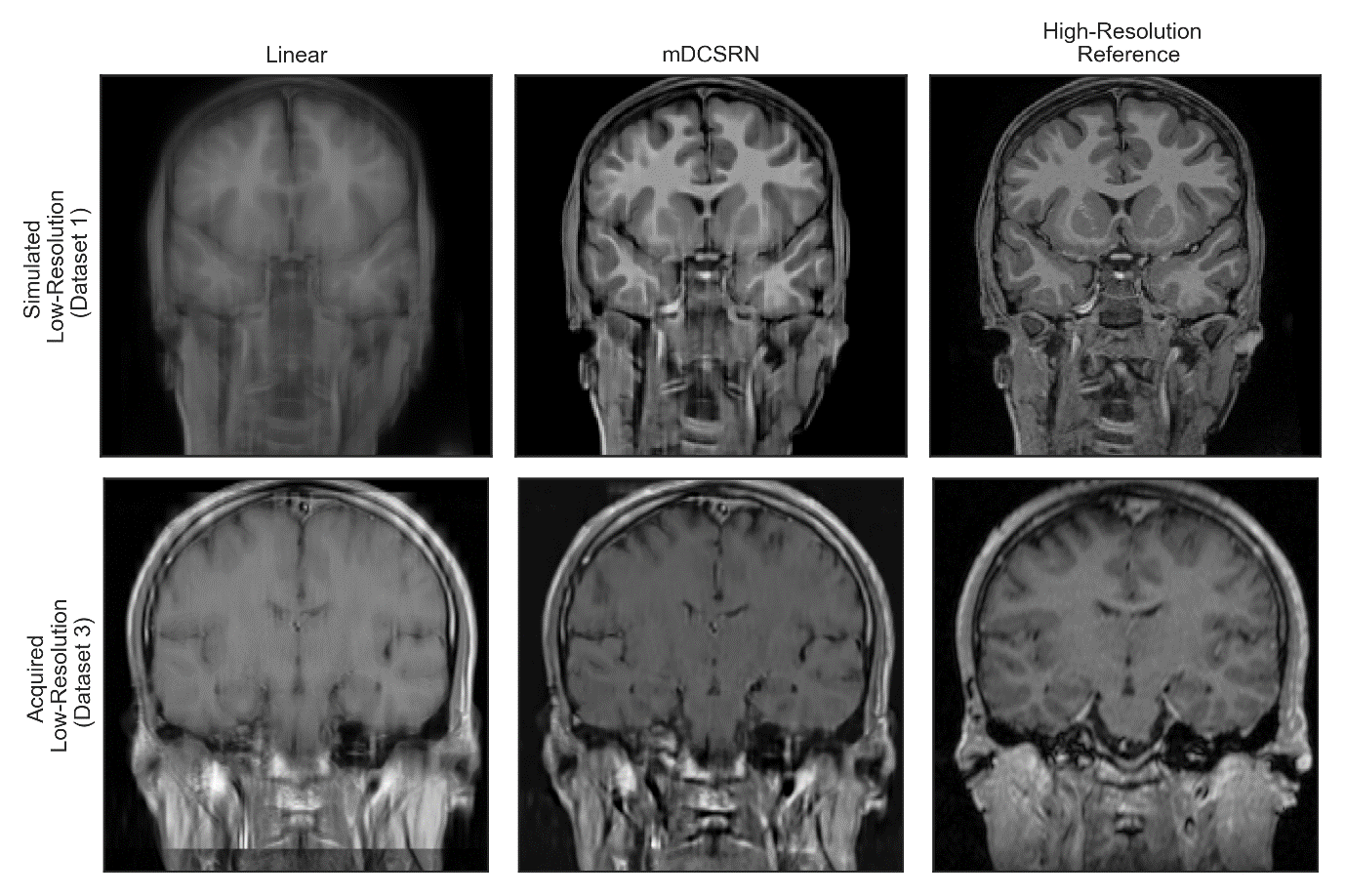


Supplementary 5 Figure 7: Coronal views (without contours) of example scan reconstructions (Linear interpolation and our model’s, mDCSRN, reconstruction), compared to the high-resolution reference image. The first row is an example from Dataset 1, where low-resolution inputs were simulated, and the second row is an example from Dataset 3, where both the input and reference were acquired MR images. These examples were taken as the images that had the worst mean distance-to-agreement for the mDCSRN model in the contours of the eyes, brainstem and hippocampi.


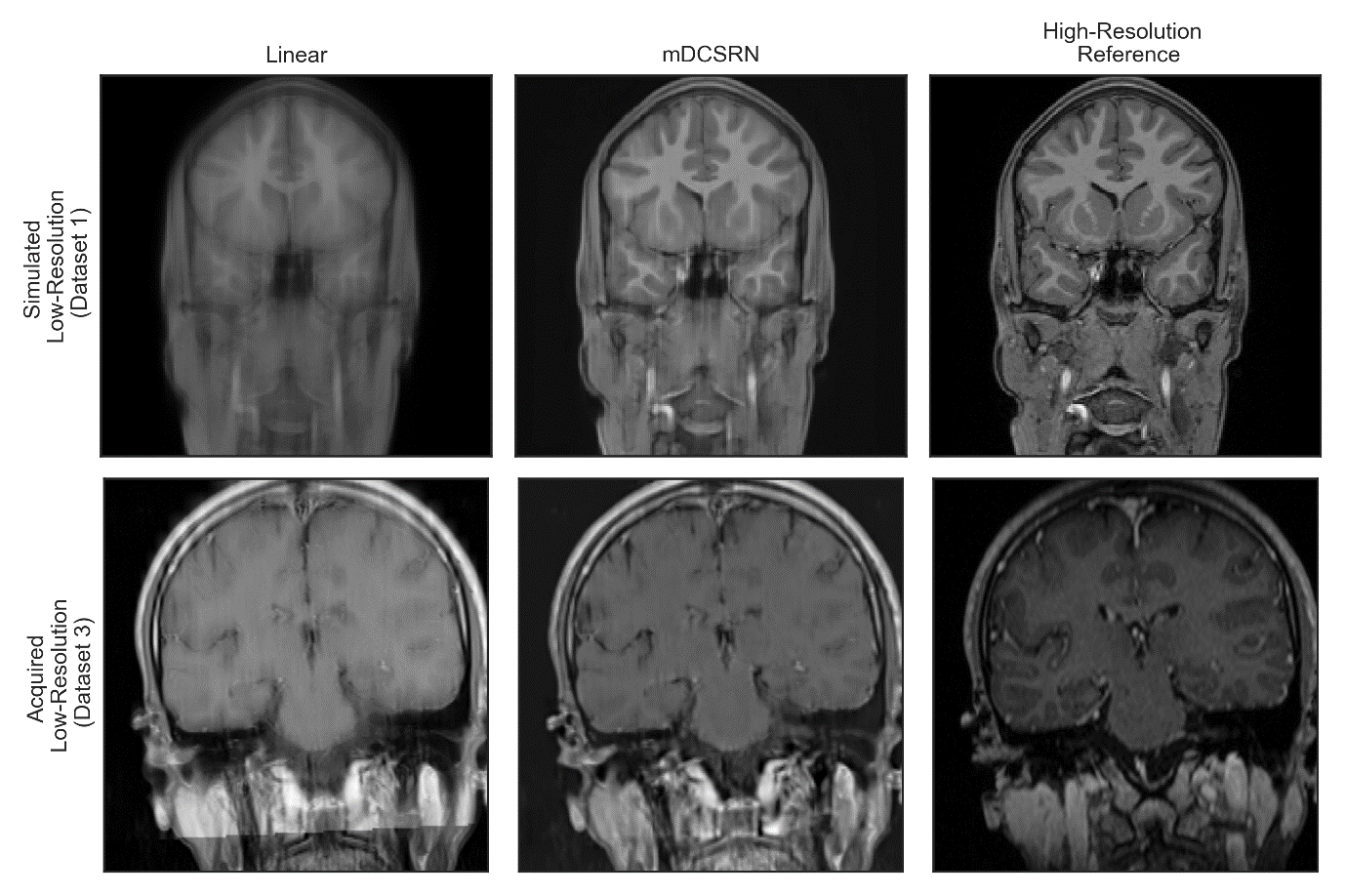


Supplementary 5 Figure 8: Coronal views (without contours) of example scan reconstructions (Linear interpolation and our model’s, mDCSRN, reconstruction), compared to the high-resolution reference image. The first row is an example from Dataset 1, where low-resolution inputs were simulated, and the second row is an example from Dataset 3, where both the input and reference were acquired MR images. These examples were taken as the images that had the best mean distance-to-agreement for the mDCSRN model in the contours of the eyes, brainstem and hippocampi.


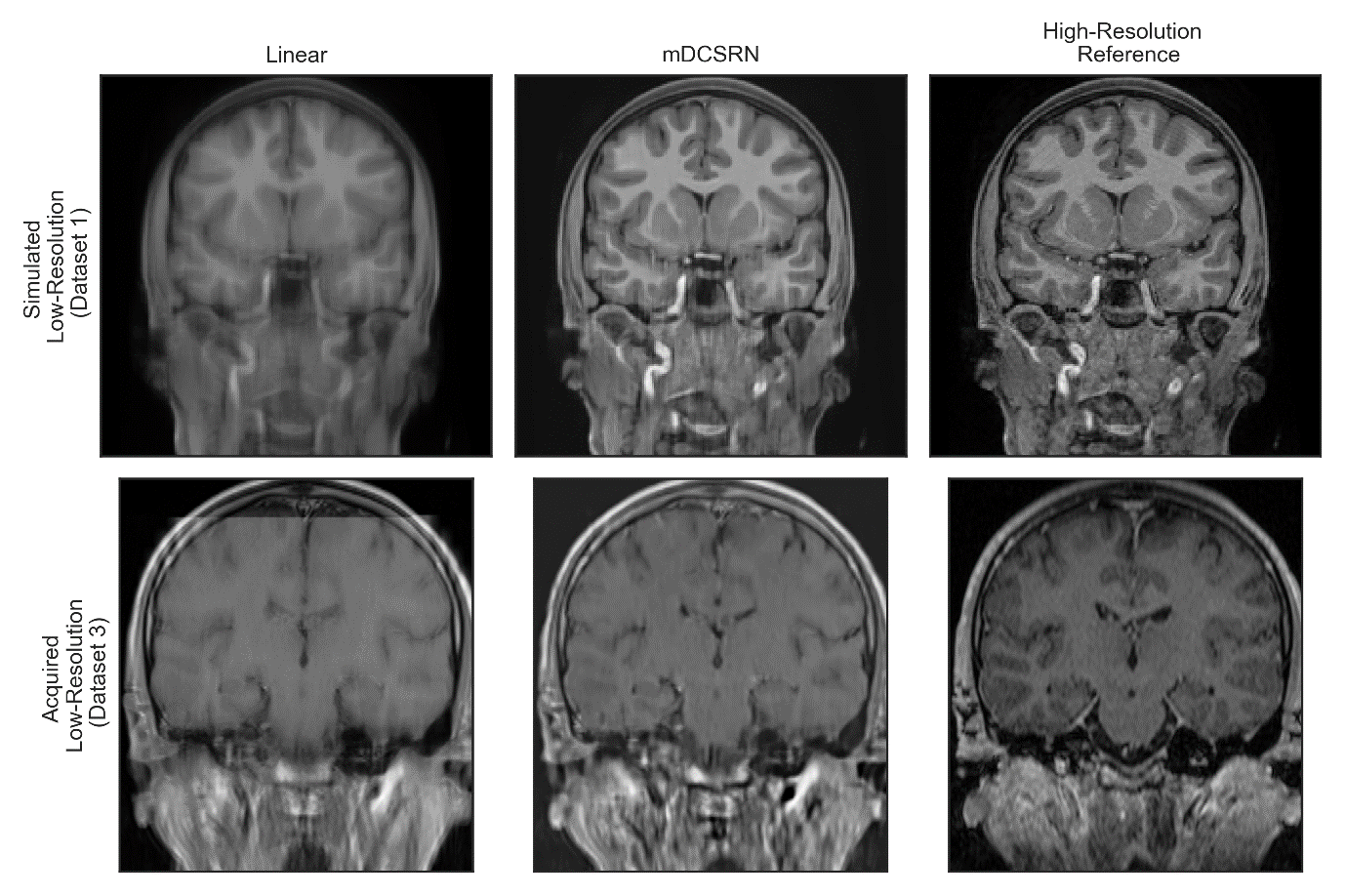


Supplementary 5 Figure 9: Sagittal views (without contours) of example scan reconstructions (Linear interpolation and our model’s, mDCSRN, reconstruction), compared to the high-resolution reference image. The first row is an example from Dataset 1, where low-resolution inputs were simulated, and the second row is an example from Dataset 3, where both the input and reference were acquired MR images. These examples were taken as the images that had the closest mean distance-to-agreement to the median of its dataset for the mDCSRN model in the contours of the eyes, brainstem and hippocampi.


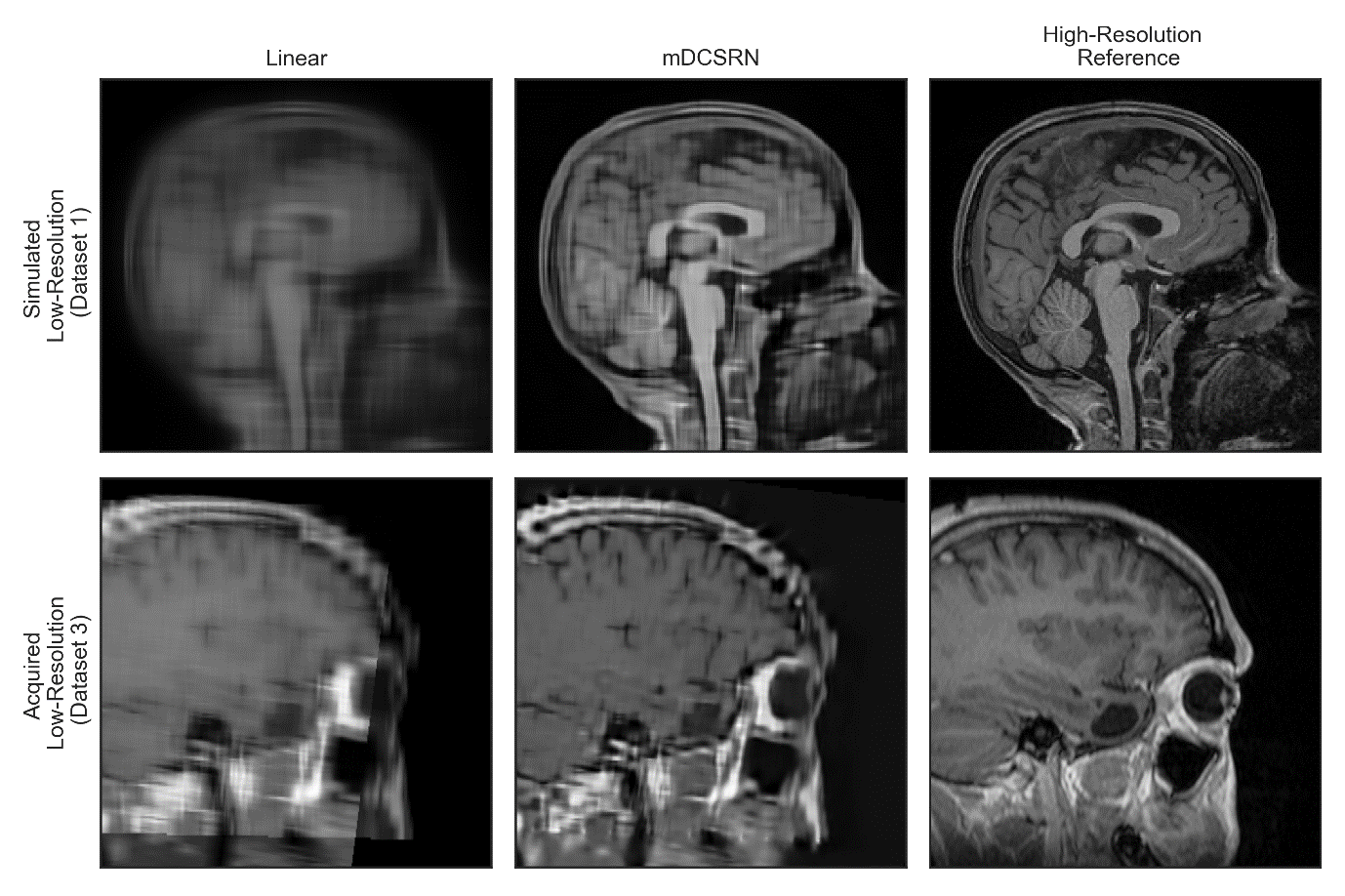


Supplementary 5 Figure 10: Sagittal views (without contours) of example scan reconstructions (Linear interpolation and our model’s, mDCSRN, reconstruction), compared to the high-resolution reference image. The first row is an example from Dataset 1, where low-resolution inputs were simulated, and the second row is an example from Dataset 3, where both the input and reference were acquired MR images. These examples were taken as the images that had the worst mean distance-to-agreement for the mDCSRN model in the contours of the eyes, brainstem and hippocampi.


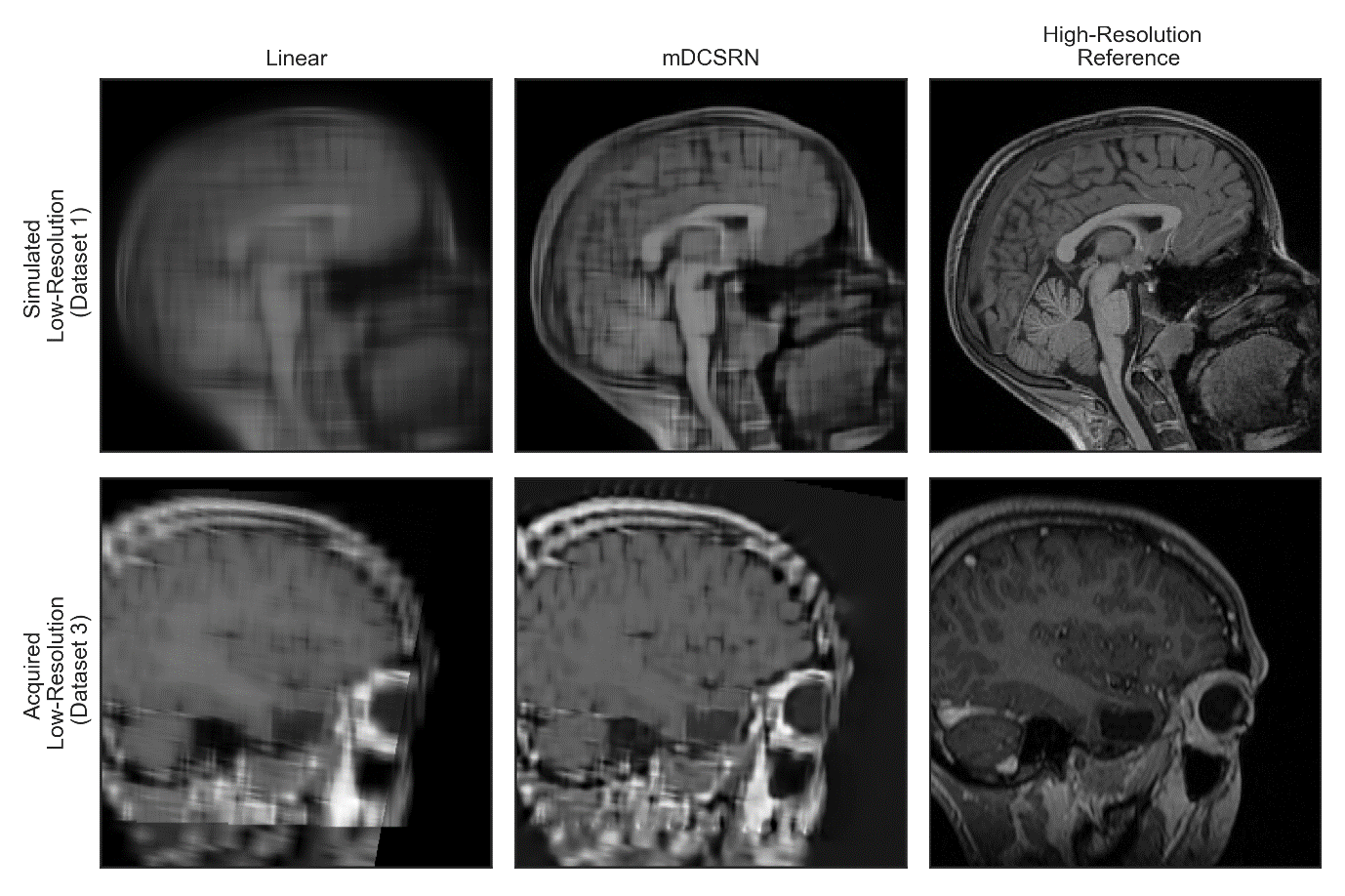


Supplementary 5 Figure 11: Sagittal views (without contours) of example scan reconstructions (Linear interpolation and our model’s, mDCSRN, reconstruction), compared to the high-resolution reference image. The first row is an example from Dataset 1, where low-resolution inputs were simulated, and the second row is an example from Dataset 3, where both the input and reference were acquired MR images. These examples were taken as the images that had the best mean distance-to-agreement for the mDCSRN model in the contours of the eyes, brainstem and hippocampi.


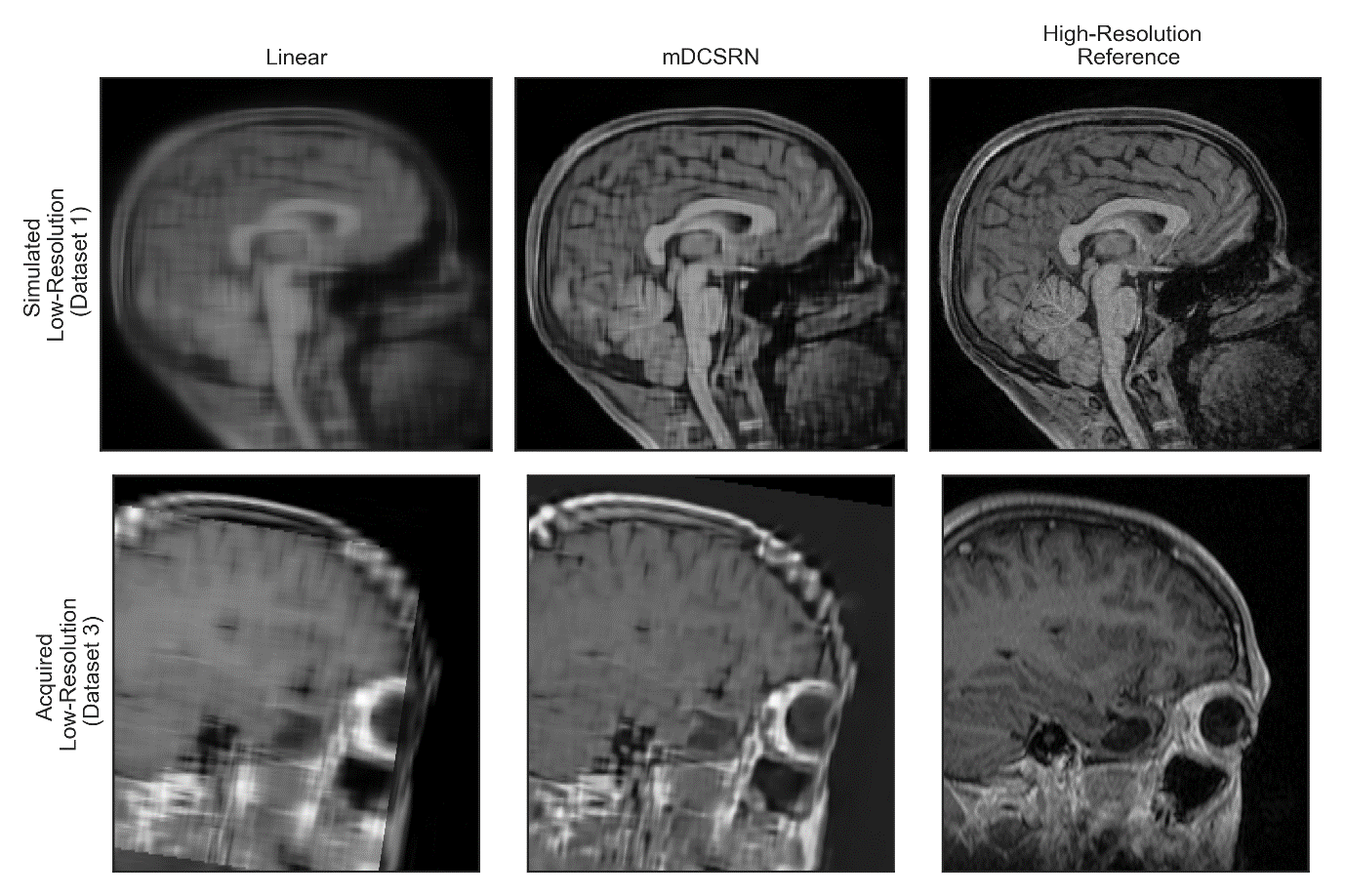

Supplement: Supplementary file 1 — Supporting Information [file MP-52-1693-s001.zip › Supplementary 5.docx]
