# Supplementary material for: A neural network to create super‐resolution MR from multiple 2D brain scans of pediatric patients
Source: Med Phys. 2024 Dec 10;52(3):1693–705. doi: 10.1002/mp.17563 (PMC11880662; doi:10.1002/mp.17563)

Supplementary 6 Figure 1: Performance of the dense convolutional neural network (mDCSRN), linear interpolation (Linear) and Lanczos interpolation (Lanczos) in terms of full-image visual similarity metrics (Peak signal-to-noise ratio, structural similarity index, mean squared error and root mean squared error) in reconstructions of high-resolution reference images from simulated low-resolution ones. Wilcoxon paired significance tests were carried out to compare the Linear and mDCSRN approaches and shown as asterisks (with one asterisk meaning p<0.05, two p<0.01 and three p<0.001, n.s. stands for non-significant).


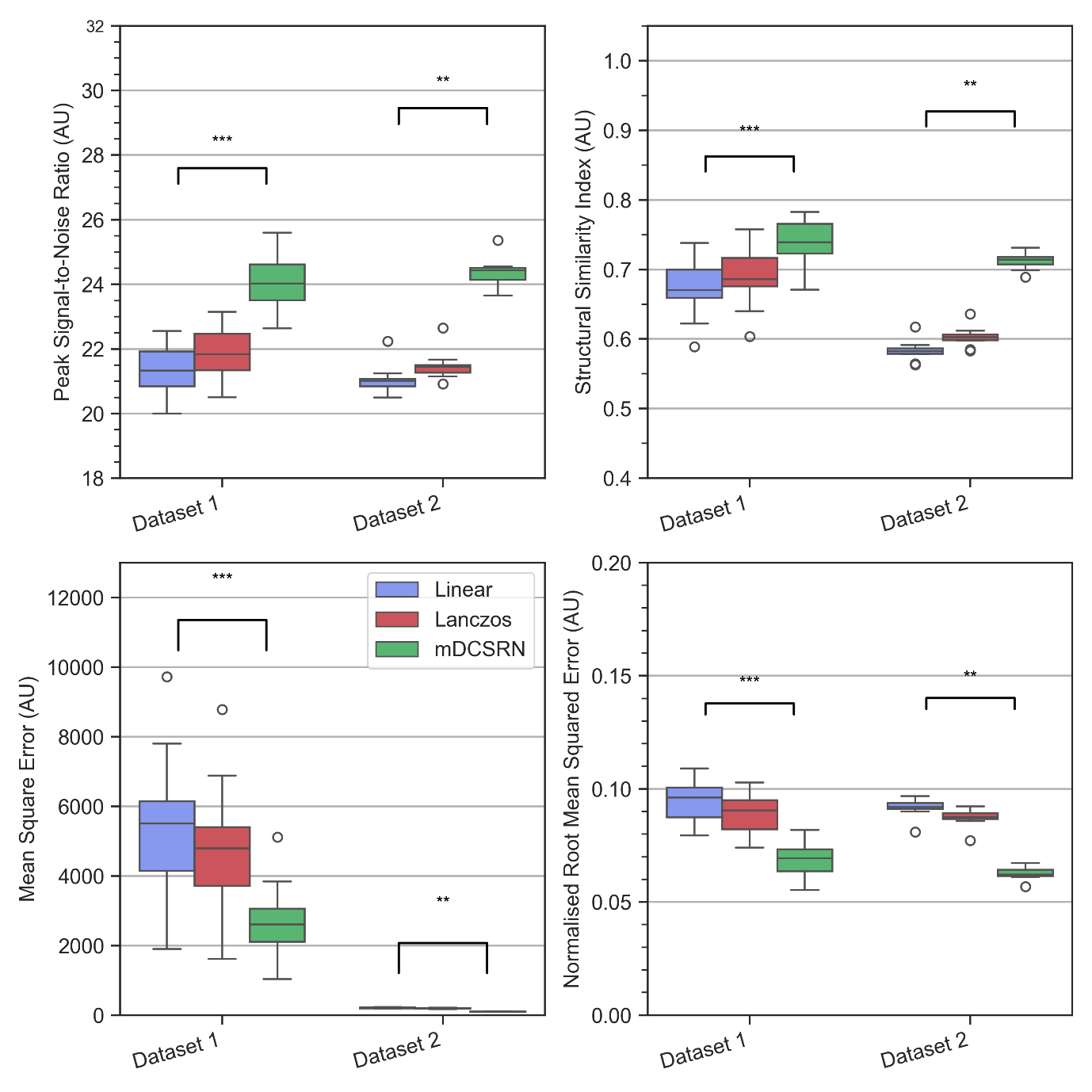

Supplement: Supplementary file 1 — Supporting Information [file MP-52-1693-s001.zip › Supplementary 6.docx]
