# Supplementary figures and images for: A neural network to create super‐resolution MR from multiple 2D brain scans of pediatric patients
Source: Med Phys. 2024 Dec 10;52(3):1693–705. doi: 10.1002/mp.17563 (PMC11880662; doi:10.1002/mp.17563)

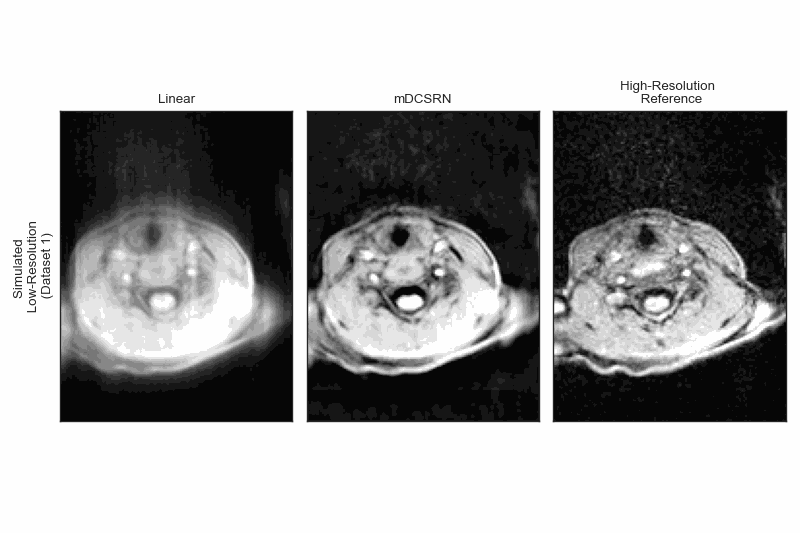

Supplement: Supplementary file 1 — Supporting Information [file MP-52-1693-s001.zip › Supplementary 4 Figure 1.gif]

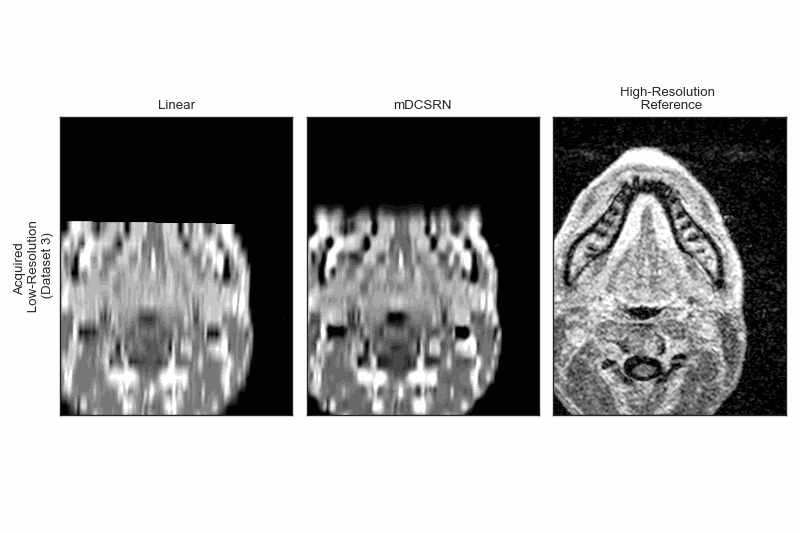

Supplement: Supplementary file 1 — Supporting Information [file MP-52-1693-s001.zip › Supplementary 4 Figure 2.gif]

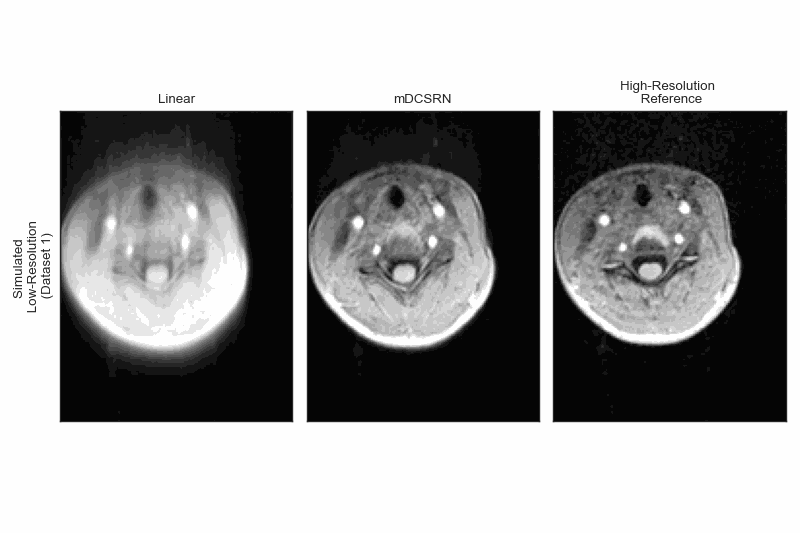

Supplement: Supplementary file 1 — Supporting Information [file MP-52-1693-s001.zip › Supplementary 4 Figure 3.gif]

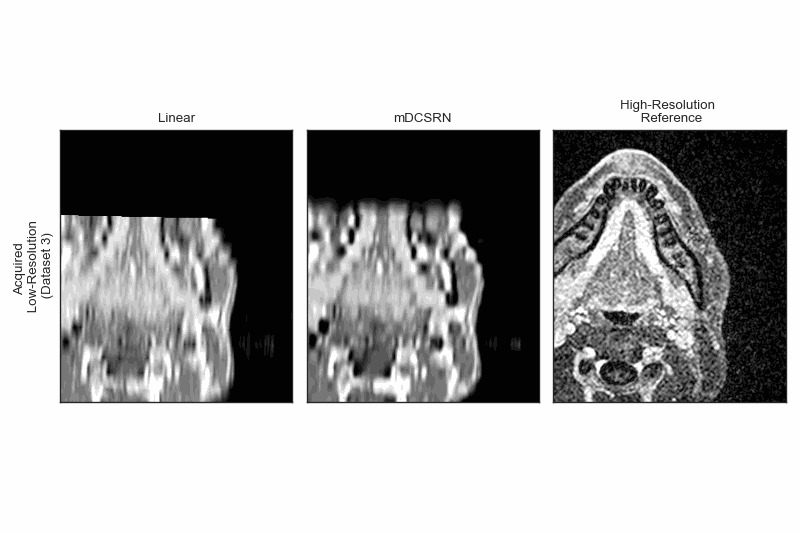

Supplement: Supplementary file 1 — Supporting Information [file MP-52-1693-s001.zip › Supplementary 4 Figure 4.gif]

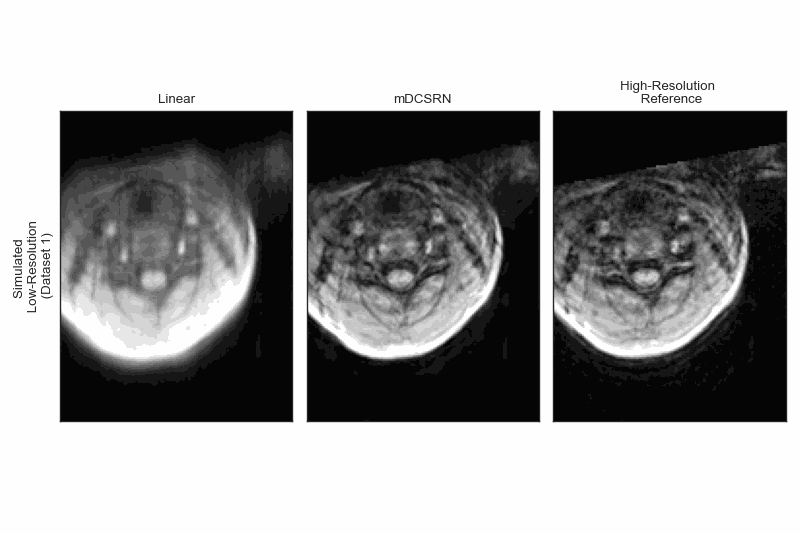

Supplement: Supplementary file 1 — Supporting Information [file MP-52-1693-s001.zip › Supplementary 4 Figure 5.gif]

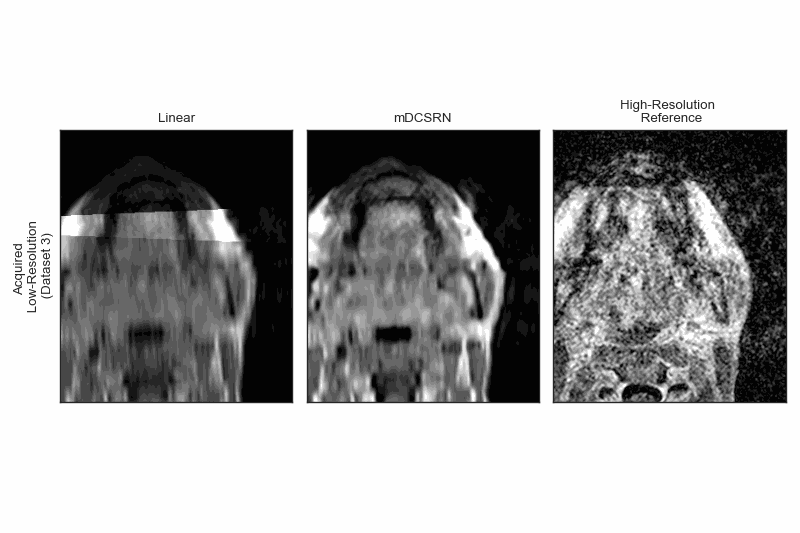

Supplement: Supplementary file 1 — Supporting Information [file MP-52-1693-s001.zip › Supplementary 4 Figure 6.gif]
